# Computational model for fetal skeletal defects potentially linked to disruption of retinoic acid signaling

**DOI:** 10.3389/fphar.2022.971296

**Published:** 2022-09-06

**Authors:** Jocylin D. Pierro, Bhavesh K. Ahir, Nancy C. Baker, Nicole C. Kleinstreuer, Menghang Xia, Thomas B. Knudsen

**Affiliations:** ^1^ Center for Computational Toxicology and Exposure (CCTE), Computational Toxicology and Bioinformatics Branch (CTBB), Office of Research and Development (ORD), U.S. Environmental Protection Agency (USEPA), Research Triangle Park, NC, United States; ^2^ Eurofins Medical Device Testing, Lancaster, PA, United States; ^3^ Scientific Computing and Data Curation Division (SCDCD), Leidos Contractor, Center for Computational Toxicology and Exposure (CCTE), USEPA/ORD, Research Triangle Park, NC, United States; ^4^ Interagency Center for the Evaluation of Alternative Toxicological Methods (NICEATM), National Toxicology Program, National Institutes of Health, Research Triangle Park, NC, United States; ^5^ Division for Pre-Clinical Innovation, National Center for Advancing Translational Sciences, National Institutes of Health, Bethesda, MD, United States

**Keywords:** New Approach Methods, retinoic acid, skeletal defects, developmental toxicity, ToxCast/Tox21

## Abstract

All-trans retinoic acid (ATRA) gradients determine skeletal patterning morphogenesis and can be disrupted by diverse genetic or environmental factors during pregnancy, leading to fetal skeleton defects. Adverse Outcome Pathway (AOP) frameworks for ATRA metabolism, signaling, and homeostasis allow for the development of new approach methods (NAMs) for predictive toxicology with less reliance on animal testing. Here, a data-driven model was constructed to identify chemicals associated with both ATRA pathway bioactivity and prenatal skeletal defects. The phenotype data was culled from ToxRefDB prenatal developmental toxicity studies and produced a list of 363 ToxRefDB chemicals with altered skeletal observations. Defects were classified regionally as cranial, post-cranial axial, appendicular, and other (unspecified) features based on ToxRefDB descriptors. To build a multivariate statistical model, high-throughput screening bioactivity data from >8,070 chemicals in ToxCast/Tox21 across 10 *in vitro* assays relevant to the retinoid signaling system were evaluated and compared to literature-based candidate reference chemicals in the dataset. There were 48 chemicals identified for effects on both *in vivo* skeletal defects and *in vitro* ATRA pathway targets for computational modeling. The list included 28 chemicals with prior evidence of skeletal defects linked to retinoid toxicity and 20 chemicals without prior evidence. The combination of thoracic cage defects and DR5 (direct repeats of 5 nucleotides for RAR/RXR transactivation) disruption was the most frequently occurring phenotypic and target disturbance, respectively. This data model provides valuable AOP elucidation and validates current mechanistic understanding. These findings also shed light on potential avenues for new mechanistic discoveries related to ATRA pathway disruption and associated skeletal dysmorphogenesis due to environmental exposures.

## Introduction

Retinoid signaling plays an important role in the patterning, differentiation and homeostasis of the developing skeletal system (Janesick et al., 2015; Ghyselinck and Duester, 2019; Mezquita and Mezquita, 2019; Knudsen et al., 2021). All-trans retinoic acid (ATRA), the biologically active form of vitamin A, is an endogenous mediator of skeletal patterning and differentiation as evidenced by various animal models of retinoid depletion or excess during pregnancy (Shannon et al., 2017: Cunningham and Duester, 2015). Sensitive gestational stages correspond to early specification of the body plan during gastrulation and subsequent organogenesis. Specifically, ATRA signaling influences spatial patterning of major body axes (e.g., anterior-posterior, dorsal-ventral), cranio-facial development, segmentation of the vertebral column, early limb outgrowth and skeletal development (Draut et al., 2019; Knudsen et al., 2021). ATRA deficiency and excess (e.g., *via* dietary retinol deficiency, exposure to excess ATRA or retinoid compounds, or functional inactivation of key nodes in retinol metabolism and/or signaling in mouse mutant models of ATRA deficiency or excess) have been shown to cause developmental skeletal defects (Cunningham and Duester, 2015; Roberts, 2020; See et al., 2008).

During mammalian development, maternal vitamin A circulates *via* retinol-binding protein, crosses the placenta (Blaner et al., 2016) and is locally synthesized into ATRA in embryonic target tissues by a two-step oxidation pathway. The first step is initiated by retinol dehydrogenase (e.g., RDH10) that oxidizes retinol to retinaldehyde (RAL), and the second by cytosolic retinaldehyde dehydrogenase (e.g., RALDH 1, 2, 3) (Metzler and Sandell, 2016; Shannon et al., 2017). Additionally, excessive ATRA buildup is inhibited in part by the reverse conversion of RAL back to retinol, a reaction catalyzed by at least one enzyme, the ATRA-inducible dehydrogenase reductase 3 (DHRS3) *via* interaction with RDH10 (Adams et al., 2014). ATRA is enzymatically degraded by cytochrome P450 monooxygenases, resulting in ATRA’s short half-life (∼1 h) (Shimozono et al., 2013; Isoherranen and Zhong, 2019). The relevant CYP26 family in embryonic patterning of ATRA gradients includes three genetically distinct isoforms (CYP26A1, CYP26B1, and CYP26C1) differing in substrate preferences for 9-cisRA and 13-cisRA (Isoherranen and Zhong, 2019). The regional patterns of RDH10/RALDH2 and CYP26A1/B1/C1 expression set up ATRA morphogenetic gradients that restrict signaling to short-range paracrine or autocrine kinematics (Shimozono et al., 2013; Teletin et al., 2017). In concert with CYP26 expression patterns, ATRA spatial and temporal gradients are developed by regional expression of dehydrogenases.

ATRA is the best known endogenous active metabolite of (retinol → RDH → RALDH → ATRA) vitamin A and is considered the cognate ligand for the retinoic acid receptor (RAR). Once inside the cell, ATRA’s signal is transduced by specific nuclear hormone receptor complexes consisting of RAR (RARA, RARB, RARG) and retinoid X receptor (RXRA, RXRB, RXRG) heterodimers, which act, in general, as ligand-activated transcription factors at a retinoic acid response element (RARE) (Mark et al., 2006; Niederreither and Dollé, 2008; Blaner et al., 2016). This complex binds DNA and induces changes in gene expression (Shannon et al., 2020) during embryonic development (Cunningham et al., 2013; Shimozono et al., 2013; Chawla et al., 2016; Schubert and Gibert, 2020). Several isoforms of each RAR and RXR exist, with distinct spatial temporal expression patterns. The diverse effects of ATRA/retinoids during normal embryonic development are mediated by the various RAR/RXR heterodimer combinations of each of these isoforms (Chambon, 1994; Kastner et al., 1994; Mark et al., 2009). The classical direct repeat (DR) for RAR/RXR binding has a 5-nucleotide spaced DR (referred to as DR5) (Balmer and Blomhoff, 2005).

In the Organisation for Economic Co-operation and Development’s (OECD’s) 2012 Detailed Review Paper (DRP) 178 focused on Endocrine Disruptor Screening Program (EDSP), the retinoic acid signaling pathway was ranked second (below PPAR signaling) among seven pathways considered to be susceptible to environmental endocrine disruption and for which relevant endpoints could be measured in a chemical testing battery (OECD 2012, 2014). Some of the most prominent adverse effects of systemic retinoid exposure, such as fetal skeletal defects, were the target for an OECD workplan to identify chemical hazards to development based on relevant *in vitro* assays (OECD, 2021).

Conserved cell signaling through ATRA-dependent gene expression has been well documented and shown to have developmental effects on most tissues (Yousefi and Azizzadeh, 2010). Prenatal development is particularly vulnerable to genetic, pharmacological, or chemical disruption of the retinoid pathway during gastrulation-organogenesis (Knudsen et al., 2021; OECD, 2021). This is especially the case for early gestation when the fundamental body plan is established, and subsequent stages when the regional pattern of specific body segments is decoded. At least 12 assays in the ToxCast/Tox21 portfolio map to molecular targets in the retinoid signaling pathway. A preliminary analysis revealed low-/submicromolar bioactivity on one or more target assays for over 100 structurally diverse ToxCast/Tox21 chemicals (e.g., conazoles, organochlorine pesticides, organotins, retinoids, and pharma compounds) suggesting that they can be used to generate models of the retinoid system and provide predictive toxicological information relevant to developmental disruption (Baker et al., 2018).

Skeletal defects are among the most prevalent adverse fetal outcomes associated with prenatal developmental toxicity in EPA’s ToxRefDB database (Knudsen et al., 2009). Menegola et al. (2021) presented an adverse outcome pathway (AOP) on disruption of ATRA signaling pathway leading to craniofacial defects. Consequences of environmental disruption of ATRA signaling pathway can lead to stage- and region-specific deficiencies for various skeletal elements (Shenefelt, 1972; Williams and Bohnsack, 2019; Zhang et al., 2015; Qin et al., 2014; Lee et al., 2012). For example, visceral and skeletal anomalies have been observed in various animal models (e.g., rat, mouse, rabbit, chick) and specifically, retinoic acid administration at embryonic day E9.5 led to hypoplasia of the branchial arches, as well as auricular and eye anomalies in mice (Glineur et al., 1999).

With tens of thousands of chemicals currently in commerce or in the environment, New Approach Methods (NAMs) including high-throughput screening/high content screening assays and computational/*in silico* models aim to identify biological pathways and chemically induced biological activity in human cells. With an emphasis on protecting susceptible populations and lifestages such as embryonic development, NAMs can develop predictive models of *in vivo* biological response that would ignite a shift from traditional animal endpoint-based testing to human pathway-based risk assessment and hazard identification, (Collins et al., 2008; US EPA, 2021). The Lautenberg Chemical Safety for the 21st Century Act (15 USC 2601 Public Law 114-182) emphasizes the need for reduction and replacement of the use of vertebrate animals in toxicity testing paradigms (2016). To this end, the ToxCast and Tox21 projects are building high-throughput screening and high content screening *in vitro* datasets, while the Toxicity Reference Database (ToxRefDB; Watford et al., 2019) is a rich database of *in vivo* data for anchoring predictive models. ToxCast and Tox21 assays cover a wide array of molecular/cellular signaling pathways (Huang et al., 2016; Thomas et al., 2019), and human stem cell line-based biomarker assays are predictive for developmental toxicity (Zurlinden et al., 2020). ToxRefDB provides prenatal developmental toxicity studies in pregnant animals (e.g., rats, mice, rabbits, and other mammals) (Knudsen et al., 2009; Sipes et al., 2011). Linking the data from all three sources supports statistical models predicting potential for adverse effects (e.g., developmental), as well as identifies potential molecular targets and cellular pathways for incorporation into virtual tissue models simulating cellular dynamics (Knudsen and Kleinstreuer, 2011; Kleinstreuer et al., 2014; Leung et al., 2016; Hutson et al., 2017). Models developed using human-relevant mechanistic information build confidence *in silico* models’ scientific reliability and relevance for regulatory decisions. A detailed predictive computational (*in silico*) signature model is therefore necessary to understand both normal embryonic skeletal development and how environmental factors may lead to a variety of skeletal developmental defects. Herein, we integrate multiple databases to develop *in silico* models that identify chemicals associated with ATRA signaling pathway disruption and fetal skeletal defects.

We used the ToxCast/Tox21 *in vitro* data with ToxRefDB *in vivo* studies in pregnant vertebrates to build a novel predictive model to identify potential developmental toxicity of chemical compounds associated with ATRA signaling pathway disruptions (RARA/B/G, RXRA/B, DR5, CYP26 Surrogates—onwards labelled CYP surrogate biomarker) and a range of skeletal defects (e.g., axial, appendicular, and cranial defects). Here, we hypothesize that a predictive model of chemical compounds associated with embryonic skeletal developmental toxicity and ATRA signaling pathway disruption will reliably provide scientifically based principles for regulatory decisions regarding chemical use related to pregnant vertebrates. This study uses NAMs data to: 1) compile data available *in vitro* ToxCast and Tox21 High-Throughput Screening assays on ATRA signaling and metabolism for n = 374 chemicals; 2) cull information from ToxRefDB and select animal studies on prenatal developmental toxicity for n = 370 ToxCast chemicals with skeletal defects identified; 3) identify the relationships of these chemicals and their association with skeletal defects using literature mining (Baker et al., 2018); 4) systematically organize the *in vitro* and *in vivo* findings to provide insight into potential molecular initiating events (MIEs) on the ATRA pathway that may lead to testable AOPs. While providing foundational weight-of-evidence, a lack of three-dimensional, dynamic biological systems limits the applicability of AOPs to systemic problems, ushering in the need for more encompassing NAMs.

## Methods

### Phenotypic data compilation

**FIGURE 1:**
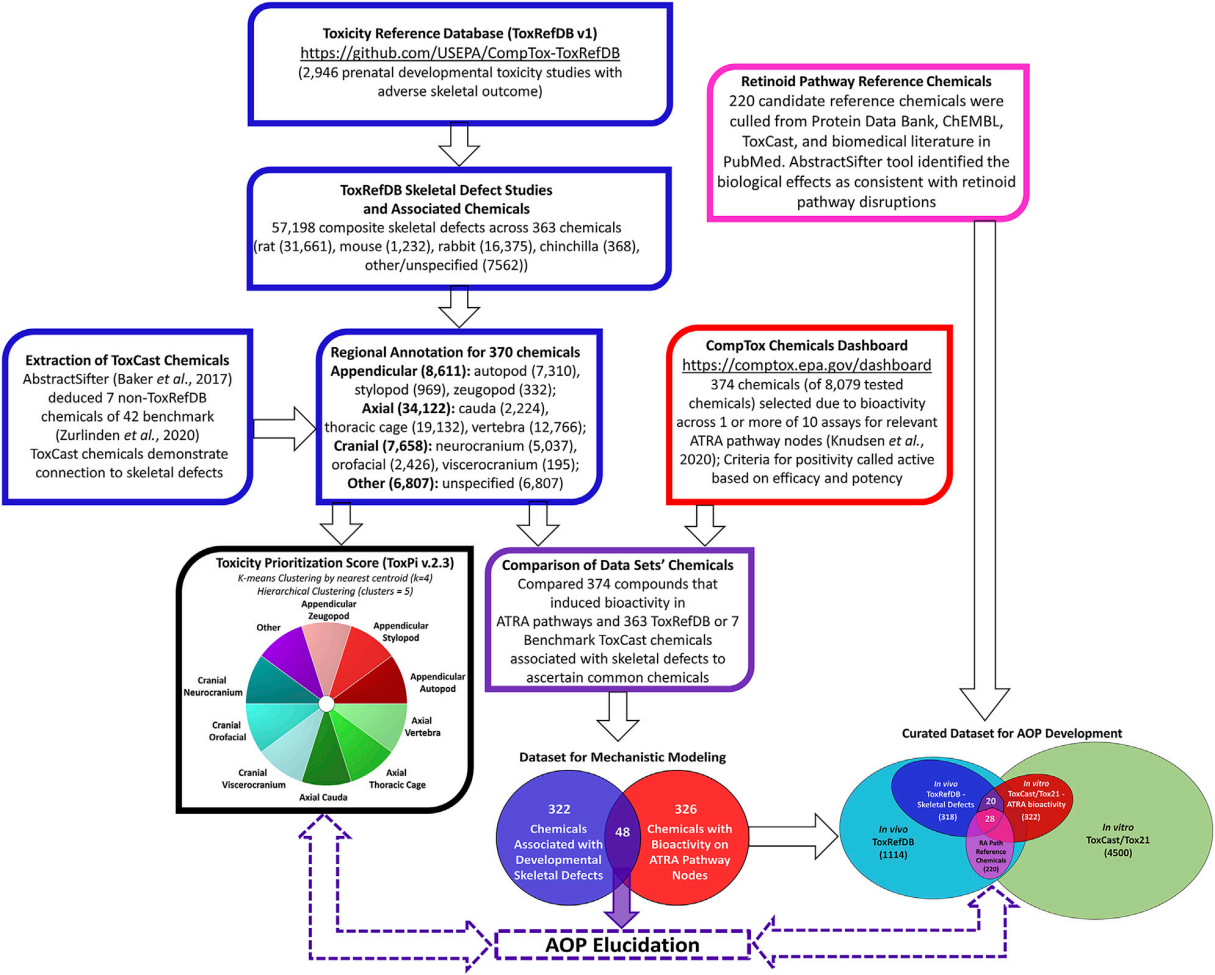
Multi-Database Pipeline for Selecting Chemicals Associated with Fetal Skeletal Defects *in vivo* and Bioactivity of ATRA Pathway Nodes *in vitro*. Chemicals associated with 57,198 composite skeletal defects were extracted from *in vivo* ToxRefDB and ToxCast Databases; defects were categorized into ten skeletal regions (blue boxes). Chemicals that induced *in vitro* bioactivity in ATRA pathways in 1 or more of 10 relevant ToxCast/Tox21 assays (red box). *In vitro* and *in vivo* data was compared, distinguishing 48 chemicals associated with both skeletal defects and bioactivity for relevant ATRA pathway nodes (purple box and Venn diagram). These chemicals potentially elucidate the role of retinoid signaling in skeletal development for AOPs and predictive toxicology applications.

Using Python v.3.3, the Toxicity Reference Database (Watford et al., 2019; ToxRefDB v.2, https://github.com/USEPA/CompTox-ToxRefDB, accessed November 2020) was mined for chemicals associated with developmental skeletal defects across 2,946 prenatal developmental toxicity studies. Python was used to categorize endpoint targets anatomically by available ToxRefDB skeletal defect annotations of Limb, Bone, Paw/Digits, and Mouth/Jaw. This resulted in the retrieval of 57,198 annotated features linked to skeletal defects (Figure 1). The distribution of skeletal defects by species included 31,661 in rats, 1,232 in mice, 16,375 in rabbits, and 368 in chinchillas. This was a composite from 363 ToxRefDB chemicals. ToxRefDB includes bone elements entered as individual targets (e.g., ribs, vertebrae, scapula, sternum, ulna, etc.) for 206 fetal bones. The database also includes the elementary description of the type of defect for each bone (e.g., absent, bent, incomplete ossification, misshapen, etc.) (Knudsen et al., 2009). Based on individual targets, data was parsed into developmental skeletal phenotypes by one of three major anatomical regions—appendicular, axial, or cranial. Anatomical development of skeletal regions has been well described for appendicular and axial skeletal regions; here, axial defects are separated into cranial and post-cranial (trunk) regions. Appendicular defects (representative of 126 bones) were classified into stylopod, zeugopod, or autopod - segments of the fore- and hindlimb skeleton. Effects on the cranial skeleton (28 bones) were classified as neurocranial, orofacial, or viscerocranial regions, and the axial defects (representative of 52 bones) were classified into thoracic cage (ribs, sternum), vertebral, or cauda regions (Knudsen et al., 2021). Lastly, a category named “other” was allocated for 7,562 unspecified developmental skeletal defects. Unspecified developmental skeletal defects are representative of data entries with elementary description of the type of skeletal defect (e.g. absent, misshapen, unossified, etc.), but that do not indicate the individual target bone (Watford et al., 2019). The inclusion of unspecified data may limit the precision of the model but provides a more complete dataset. Zurlinden et al. (2020) highlighted 42 ToxCast chemicals that are extensively used by researchers in developmental toxicology non-animal models. A few of these well-annotated developmental toxicants were found in ToxCast but not included in ToxRefDB. We searched PubMed using the Abstract Sifter semi-automated literature mining tool (Baker et al., 2017) to derive information from scientific publications related to these 42 chemicals, and identified skeletal defects associated with 7 of these substances. Combined, ToxRefDB and ToxCast chemicals linked to developmental defects in 10 skeletal regions as provided in Figure 1.

### Bioactivity data compilation

In the U.S. Environmental Protection Agency’s ToxCast high-throughput screening program over 4,000 chemicals have been tested for bioactivity for over 1,400 molecular targets/features accessed through InVitroDB v.3.1 at https://epa.figshare.com/articles/dataset/Previously_Published_ToxCast_Data/6062551 in November 2020 (Judson et al., 2016; Richard et al., 2016). The breadth of ToxCast assays include biochemical assays, human cells, mouse embryonic stem cells and zebrafish development platforms (Thomas et al., 2019; Parish et al., 2020; Marty et al., 2022). In the Tox21 project, about 8,500 chemicals were screened, including the over 4,000 ToxCast chemicals. Our project mined a combined 10 assays (Table 1) from ToxCast and Tox21 (Chen et al., 2016) for chemicals that demonstrated bioactivity for relevant ATRA nodes (RARs, DR5, RXRs, and CYP surrogate biomarker). CYP1A1 and CYP2C8 were selected as CYP surrogate biomarker, due to the absence of CYP26 assays in ToxCast and Tox 21 (Baker et al., 2022). Importantly, both CYP surrogate biomarker’ assays are biochemical. The binding region of CYP26 is similar to CYP2C8. When CYP2C8 inhibitors were tested against CYP26A1, there was a statistically significant correlation between CYP26A2 and CYP2C8 IC_50s_ (Foti et al., 2016a; Foti et al., 2016b). The enzyme CYP1A1 is also recorded as metabolizing retinoic acid (Lampen et al., 2000). NovaScreen human unspecified cytochrome P450, family 1, subfamily A, polypeptide 1 assay (NVS_ADME_hCYP1A1) and NovaScreen human unspecified cytochrome P450, family 2, subfamily C, polypeptide 8 assay (NVS_ADME_hCYP2C8) assay data was combined as CYP surrogate biomarker, using the lowest AC_50_ if redundant. NovaScreen human unspecified retinoic acid receptor, alpha assay (NVS_NR_hRARa_Agonist) data was combined with Attagene human HepG2 retinoic acid receptor, alpha assay (ATG_RARa_TRANS_up) information (no overlap in data occurrences) to compose RARA bioactivity figures utilized (Table 1). While these assays have different dynamic range, both test for increases in RARA bioactivity, and the range of concentrations for which activity were selected were limited by a threshold (<10 µM) chosen for both environmental relevance and specificity to avoid generalized cell stress responses (Judson et al., 2016). Along the ATRA signaling pathway, in both databases, relevant assays were measured for interactions between chemicals and receptors and enzymes as molecular targets or assessed downstream effects on reporter gene activity. Chemicals were selected from these assays based upon their potency, half-maximal activity concentrations (AC_50s_), and efficacy in one or more related assays. These chemicals were then compared to the list of ToxRefDB/ToxCast chemicals associated with skeletal phenotypic defects.

**TABLE 1:** | The 10 ToxCast and Tox21 assays that tested over 8,079 chemicals for bioactivity for relevant ATRA.

Assay	Gene examined	Assay description
ATG_DR5_CIS_up	DR5	Cis-reporter assay related to RARE activation
ATG_RARa_TRANS_up	RARA	Reporter for RARA transactivation
ATG_RARb_TRANS_up	RARB	Reporter for RARB transactivation
ATG_RARg_TRANS_up	RARG	Reporter for RARG transactivation
ATG_RXRa_TRANS_up	RXRA	Reporter for RXRA transactivation
ATG_RXRb_TRANS_up	RXRB	Reporter for RXRB transactivation
NVS_ADME_hCYP1A1	CYP1A1	Biochemical reporter, loss of activity related to human CYP1A1
NVS_ADME_hCYP2C8	CYP2C8	Biochemical reporter, loss of activity related to human CYP2C8
NVS_NR_hRARa_ Agonist	RARA	Biochemical, loss of activity related to RARA agonist liganding
TOX21_RAR_LUC_ Agonist	RARA	Reporter for RARA transactivation related to retinol signaling pathway (RSP)

### Data analysis and visualization

**FIGURE 2:**
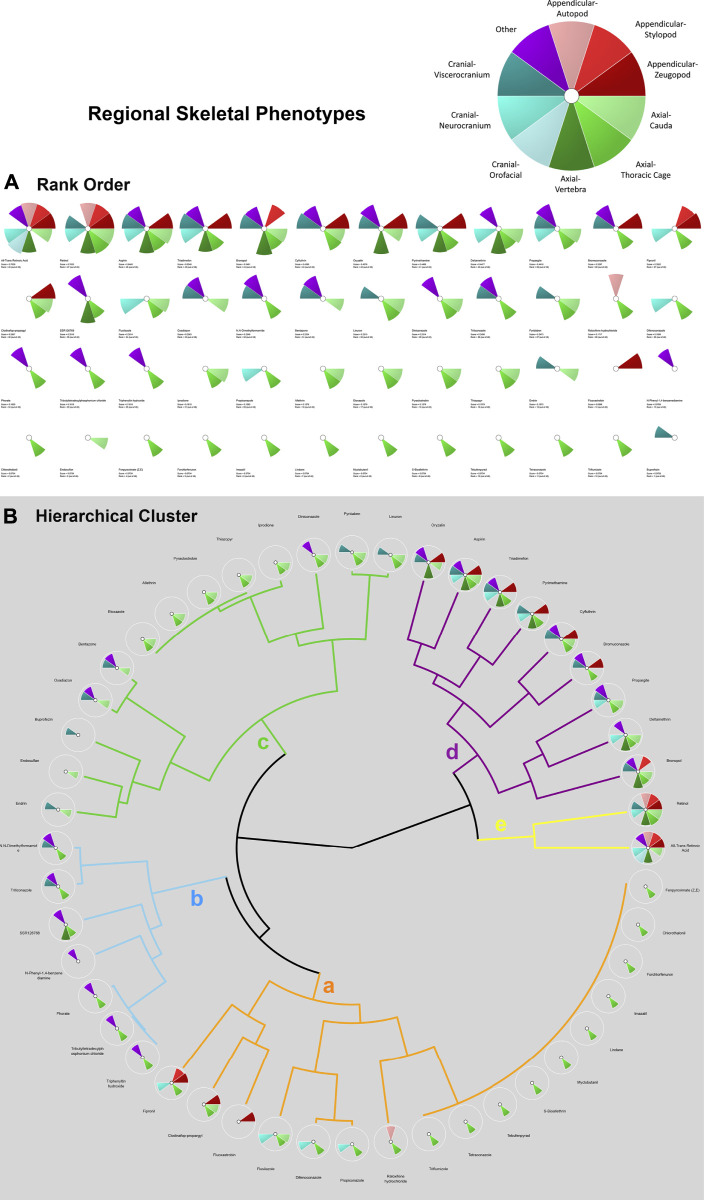
ToxPi Rank Order **(A)** and Hierarchical Clustering **(B)** results for 48 chemicals associated with adverse skeletal outcomes (ASOs). ASOs include autopod, stylopod, zeugopod, cauda, thoracic cage, vertebra, orofacial, neurocranium, viscerocranium, and other. Most potent chemicals associated with the greatest amount of target disruption have the largest ToxPis and highest ToxPi rank (48). Skeletal defects were categorized into three skeletal phenotypes and specified regions and an unspecified region as delineated in the ToxPi key **(A)**. Appendicular regions are shades of red, axial regions are shades of green, cranial regions are blues, and unspecified skeletal areas are purple. Skeletal Defects Hierarchical Clustering for 48 Chemicals: ‘Clusters a-e’ represent varying skeletal phenotype alterations associated with up to 48 chemicals of interest. Clusters with chemical ToxPis associated with similar skeletal defect composition. ‘Cluster a was primarily thoracic cage defects with occurrences of neurocranium and appendicular defects. A ToxPi rank of 1 is least potent, while a score of 48 is a highly potent associated with significant skeletal defects. Rank order of ‘Cluster a’ chemicals is 1. Fenpyroximate (Z,E), 2. Chlorothalonil, 3. Forchlorfenuron, 4. Imazalil, 5. Lindane, 6. Myclobutanil, 7. S-Bioallethrin, 8. Tebufenpyrad, 9. Tetraconazole, 10. Triflumizole, 11. Raloxifene hydrochloride, 12. Propiconazole, 13. Difenoconazole, 14. Flusilazole, 15. Fluoxastrobin, 16. Clodinafop-propargyl, and 17. Fipronil. ‘Cluster b’ was dominated by unspecified and thoracic cage defects, with 2 occurrences of viscerocranial defects. Rank order of ‘Cluster b’ chemicals is 18. Triphenyltin hydroxide, 19.Tributyltetradecylphosphonium chloride, 20. Phorate, 21. N-Phenyl-1,4 benzenediamine, 22. SSR126768, 23. Triticonazole, and 24. N,N-Dimethylformamide. ‘Cluster c’ is dominated by axial defect with occurrences of viscerocranial and unspecified defects. Rank order of ‘Cluster c’ chemicals is 25. Endrin, 26. Endosulfan, 27. Buprofezin, 28. Oxadiazon, 29. Bentazone, 30. Etoxazole, 31. Allethrin, 32. Pyraclostrobin, 33. Thiazopyr, 34. Iprodione, 35. Diniconazole, 36. Pyridaben, and 37. Linuron. ‘Cluster d’ possesses zeugopod phenotype, the full axial zone, and neurocranial and viscerocranial regions defects. Rank order of ‘Cluster d’ chemicals is 38. Oryzalin, 39. Aspirin, 40. Triadimefon, 41. Pyrimethamine, 42. Cyfluthrin, 43. Bromuconazole, 44. Propargite, 45. Deltamethrin, 46. Bronopol. The most potent chemicals of Retinol (47) and ATRA (48) were strongly associated with multiple appendicular, axial, and cranial phenotypic defects.

**FIGURE 3:**
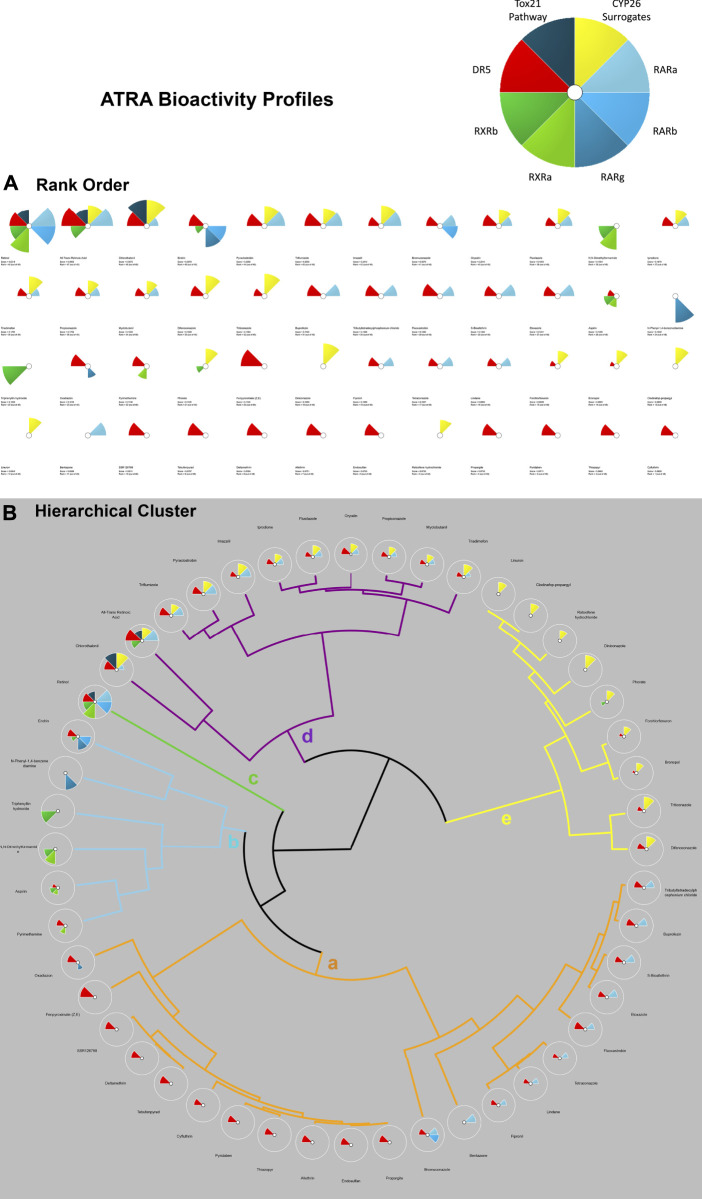
ToxPi Rank Order **(A)** and Hierarchical Clustering **(B)** results for 48 Chemicals associated with disruption of targets on the ATRA signaling pathway. The ToxPi for ATRA signaling pathway disruption target types of RARs are shades of blue, while DR5 is red, RXRs are shades of green, CYP surrogate biomarker are yellow, Tox21 is a dark teal. Targets of interest include RARA/B/C, RXR A/B, DR5, Retinol Signaling Pathway (Tox21), CYP surrogate biomarker. Most potent chemicals associated with the greatest amount of ATRA signaling target disruption have the largest ToxPis and highest ToxPi rank (48). A ToxPi rank of 1 is least potent, while a score of 48 is a highly potent associated ATRA bioactivity. Hierarchical Clustering for 48 Chemicals In Vitro Activity on ATRA Signaling Pathway Targets: ‘Clusters a-e’ represent varying targets disrupted on the ATRA signaling pathway by up to 48 chemicals of interest. Clusters of chemical ToxPis associated with similar target disruption composition. ‘Cluster a’ is dominated by DR5 and RARA disruption. Rank order of ‘Cluster a’ chemicals 1. Tributyltetradecyl-phosphonium chloride, 2. Buprofezin, 3. S-Bioallethrin, 4. Etoxazole, 5. Fluoxastrobin, 6. Tetraconazole, 7. Lindane, 8. Fipronil, 9. Bentazone, 10. Bromuconazole, 11. Propargite, 12. Endosulfan, 13. Allethrin, 14. Thiazopyr, 15. Pyridaben, 16. Cyfluthrin, 17. Tebufenpyrad, 18. Deltamethrin, 19. SSR126768, 20. Fenpyroximate (Z,E), and 21. Oxadiazon. ‘Cluster b’ possesses RXRA, RXRV, DR5, RARB/G activity. Rank order of ‘Cluster b’ chemicals 22. Pyrimethamine, 23. Aspirin, 24. N,N-Dimethylformamide, 25.Triphenyltin hydroxide, 26. N-Phenyl-1,4-benzenediamine, and 27. Endrin. ‘Cluster c’ has instances of disruption from every category of target (RARA, RXR, DR5, and RSP). Rank order of ‘Cluster c’ chemical is 28. Retinol. ‘Cluster d’ has a consistent significant CYP surrogate biomarker’ disruption, coupled with RARA and DR5 disruption. Rank order of ‘Cluster d’ chemicals 29. Chlorothalonil, 30. All-Trans Retinoic Acid, 31. Triflumizole, 32. Pyraclostrobin, 33. Imazalil, 34. Iprodione, 35. Flusilazole, 36. Oryzalin, 37. Propiconazole, 38. Myclobutanil, and 39. Triadimefon. ‘Cluster e’ primarily provides ToxPis with CYP26 disruption, with a few cases of DR5 activation and a singular case of RXRG disruption. Rank order of ‘Cluster e’ chemicals 40. Linuron, 41. Clodinafop-propargyl, 42. Raloxifene hydrochloride, 43. Diniconazole, 44. Phorate, 45. Forchlorfenuron, 46. Bronopol, 47. Triticonazole, and 48. Difenoconazole.

In addition to the multi-database comparison to identify chemicals associated with skeletal defects as well as ATRA signaling pathway disturbances, ToxCast and ToxRefDB chemicals associated with developmental skeletal defects were prioritized for potency and regional effects through the Toxicological Priority Index (ToxPi™) (ToxPi; Marvel et al., 2018). ToxPi is a software that objectively conducts chemical prioritization *via* integration across multiple information domains and sources of evidence The “ToxPi visualization” portrays the relative magnitudes of environmental hazards (e.g. exposure of environmental chemicals). Data is transformed into transparent, visual rankings (Reif et al., 2010). A ToxPi for chemicals associated with ATRA pathway disturbances was formulated for further comparison between the two (*in vivo vs*. *in vitro*) endpoints (Figure 3). Separately, for each chemical, a ToxPi was created that was broken into 10 slices, each slice representative of the 10 potential regional skeletal defects (Figure 2). Separately, Slices are proportionate to the number of each effect caused by a particular compound, which the overall potential ToxPi value equaling one. The width of each slice signifies the number of data points per defect. The larger the radius of the slice from the origin, the greater the chemical’s potency, with length representing the overall degree of hazard (National Academy of Sciences, 2014). This approach allows for a multidimensional analysis of the relative potency (*in vivo* experiments), chemical properties (bioavailability), and perturbation score (pathways). A dimensionless index score, ToxPi, is calculated based on the combination of all the data sources, and then compounds are ranked to prioritize which chemicals should be tested for future toxicological testing, compare toxicity, and identify similarities in predicted compound activity.

For k-means clustering, ToxPi employs a Java port of the R function for means. The k-means clustering module uses agglomerative clustering to organize ToxPi profiles into clusters (collection of data points) based upon similarity and discover underlying patterns. In ToxPi, k-means clustering uses the centroid to parse the chemicals rather than default sorting by overall priority score (rank). In short, k-means clustering identifies k number of centroids, assigns each data point to the nearest cluster, simultaneously keeping the centroids as small as possible. The major sources of organization were then visualized on a coordinate field by Principal Components Analysis (PCA) (Marvel et al., 2018). This function uses the “Hartigan-Wong” algorithm to execute the clustering. For all k-means clustering procedures, the algorithm was run (nStart) at the default of 15 starting cluster locations, as consistency of results was visible through such a procedure. Effective k-means clustering results in smaller within group sum of squares, which can be achieved by sound selection of the seed of the random number generator, and specified replication (Marvel et al., 2018, Manual v. 2.3). The k-means algorithm grouped similar ToxPi data points together based on distance from centroids, the center of these clusters. These groupings identified underlying patterns. For k-means clustering chemicals involving ATRA signaling pathway activated genes, 4 centroids were designated based upon biological reasoning that the number of clusters needed is one less than the total number of the relevant molecular targets involved in the assays. This allows for the statistical subtraction of one degree of freedom; similarly, 4 clusters were employed for k-means analysis of chemicals associated with ATRA pathway target (Figure 3). For the skeletal defects ToxPi, data points were assigned to one of 5 clusters (Figure 2). Homogeneous subgroups within the data are identified such that data points in each cluster are as similar as possible according to a similarity measure of skeletal region defects or ATRA signaling pathway node disturbance for the respective k-means cluster.

The Hierarchical Clustering module provides options for organizing ToxPi profiles into clusters based upon similarity, rather than the default sorting by overall priority score (rank). The cluster dendrograms are drawn using an agglomerative hierarchical clustering technique. With ToxPi profiles for individual chemicals, data points are treated as individual clusters. Then with additional iterations, similar clusters merge, until there is one cluster formed of similar data point (Reif et al., 2010; Grimm et al., 2016; Marvel et al., 2018). The Ward.D2 hierarchical clustering tool was utilized to minimize variance and visualized with the ToxPi 2.3 software. The chemicals identified as associated with skeletal defects and ATRA pathway disturbances were selected and displayed in a heatmap using the chemical AC_50_ per each of the 8 targets (pheatmap function in R).

## Results

### Initial workflow

ToxRefDB *in vivo* and ToxCast/Tox21 *in vitro* data was compared, identifying 48 chemicals (Table 2) that were associated with both skeletal defects and bioactivity (<10 µM) for relevant ATRA pathway nodes (Figure 1), purple box and Venn diagram). These chemicals have the potential to elucidate the role of retinoid signaling in skeletal development for hypothesizing and testing AOPs and supporting predictive toxicology applications. The 48 chemicals can also be found in the accompanying Abstract Sifter tool organized by target on the AbstractSifter_Retinoid sheet (Supplementary Material S1), in a simple list on the Notes sheet showing the chemical-specific association with nine skeletal defects phenotypes, and on the Landscape sheet, shown with sample toxicity-related queries and resulting article counts. The Abstract Sifter’s Landscape sheet results show that, of the 48 chemicals, retinol and ATRA are associated with the highest number of articles (293 and 247, respectively) describing skeletal defects-specific toxicity (S1). Forty-one of the 48 chemicals on the Landscape sheet have some connections to citations about skeletal defects-specific phenotypes. Seven chemicals (bromuconazole, buprofezin, fenpyroximate (Z,E), linuron, *N*-phenyl-1,4-benzenediamine, tebufenpyrad and tributyltetradecylphosphonium chloride) do not have literature associating them with skeletal defects.

**TABLE 2:** | 48 chemicals associated with skeletal defects and ATRA Signaling Pathway Disruption (ToxRefDB and ToxCast/Tox21).

48 chemicals analyzed			
DTXSID	Name	DTXSID	Name
DTXSID8035180	Allethrin	DTXSID2024163	Linuron
DTXSID7021239	All-Trans Retinoic Acid	DTXSID8024315	Myclobutanil
DTXSID5020108	Aspirin	DTXSID6020515	N,N-Dimethylformamide
DTXSID0023901	Bentazone	DTXSID7025895	N-Phenyl-1,4-benzenediamine
DTXSID9032531	Bromuconazole	DTXSID8024238	Oryzalin
DTXSID8024652	Bronopol	DTXSID3024239	Oxadiazon
DTXSID8034401	Buprofezin	DTXSID4032459	Phorate
DTXSID0020319	Chlorothalonil	DTXSID4024276	Propargite
DTXSID6032354	Clodinafop-propargyl	DTXSID8024280	Propiconazole
DTXSID5035957	Cyfluthrin	DTXSID7032638	Pyraclostrobin
DTXSID8020381	Deltamethrin	DTXSID5032573	Pyridaben
DTXSID4032372	Difenoconazole	DTXSID9021217	Pyrimethamine
DTXSID2040363	Diniconazole	DTXSID1034181	Raloxifene hydrochloride
DTXSID1020560	Endosulfan	DTXSID3023556	Retinol
DTXSID6020561	Endrin	DTXSID2039336	S-Bioallethrin
DTXSID8034586	Etoxazole	DTXSID0047379	SSR126768
DTXSID2032550	Fenpyroximate (Z,E)	DTXSID0034223	Tebufenpyrad
DTXSID4034609	Fipronil	DTXSID8034956	Tetraconazole
DTXSID2034625	Fluoxastrobin	DTXSID1032488	Thiazopyr
DTXSID3024235	Flusilazole	DTXSID3023897	Triadimefon
DTXSID1034634	Forchlorfenuron	DTXSID9034997	Tributyltetradecylphosphonium chloride
DTXSID8024151	Imazalil	DTXSID2032500	Triflumizole
DTXSID3024154	Iprodione	DTXSID1021409	Triphenyltin hydroxide
DTXSID2020686	Lindane	DTXSID0032655	Triticonazole

### ToxPi, K-means clustering, and hierarchical clustering

**FIGURE 4:**
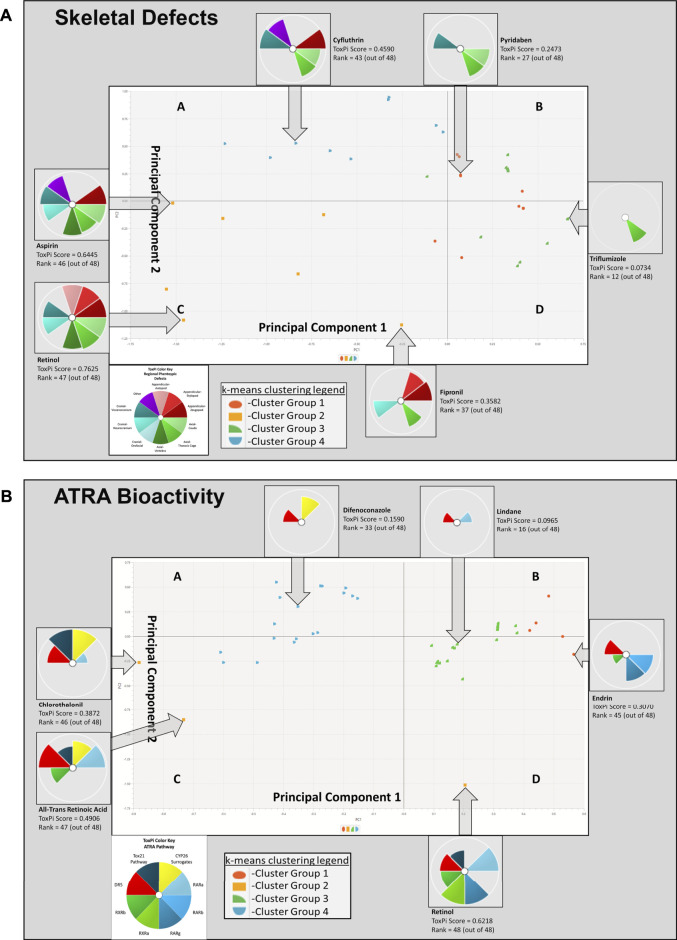
K-means clustering of Skeletal Defects **(A)** and ATRA Bioactivity **(B)** for 48 chemicals. In image A, skeletal defects examined include autopod, stylopod, zeugopod, cauda, thoracic cage, vertebra, orofacial, neurocranium, viscerocranium, and other. Principal Component 1 (PC1), the x-axis, identifies variability in the 48 model chemicals, indicating heterogeneity in the skeletal response. Moving from left to right, a broader range of phenotypic changes are found, and then the array of skeletal responses diminishes with horizontal progression. The left side PC1 demonstrates less specificity, while the right side has more specificity (i.e., single thoracic cage slice). This is demonstrable by the leftmost cluster group 2 having the most *in vivo* ToxPi regional phenotype slices at upwards of 8 slices (e.g., retinol, aspirin, etc.), while the farthest right cluster group 3 has solely 1 slice of specified regional phenotypic change (e.g., triflumizole). PC2, y-axis, demonstrates phenotypic separation as deficiencies transition from autopod to zeugopod and upward phenotypic changes are then found in the axial cauda, and thereafter moving downward there is an increase in neurocranial responses. Moving further left and upward along PC2 viscerocranial defects increase. Through PC2, a trend in axial phenotypic changes occur first, followed by an increase in cranial defects. In image B, k-means clustering ATRA Signaling Pathway for 48 chemicals: Disrupted activity in the following targets are of interest: RARA/B/C, RXR A/B, DR5, RSP (Tox21), CYP surrogate biomarker. PC1 and PC2 consistently demonstrate that DR5 is the most sensitive target, with interruption occuring in all k-means quadrants to some degree. Overall CYP surrogate biomarker, RARA, and DR5 are most disrupted along the ATRA signaling pathway in the 10 assays examined.

K-means clustering of skeletal defects provided insight into the most sensitive phenotypic changes following chemical exposure. Analysis of the skeletal defects K-means clustering plot (Figure 4A) indicates that Principal Component 1 (PC1), the *x*-axis, identifies variability in the 48 model chemicals, denoting the heterogeneity of the skeletal response. Summarily, the left side of Figure 4A PC1 demonstrates less specificity with 8 phenotypic regions (affected by, e.g., retinol, aspirin) represented, while the right side has more specificity as indicated by the individual thoracic cage slice (affected by, e.g., triflumizole). PC2, *y*-axis, provides the specificity of the regional phenotype based on chemical. Through PC2, a trend in axial phenotypic changes (thoracic cage, cauda, and then vertebral) occur first, followed by an increase in cranial defects (progressively neurocranium, viscerocranium, and then orofacial regions). Thoracic cage alterations occur without other phenotypic changes preceding its own. Again, looking at the systemic implications of these diagrams, thoracic cage formation is tied to homeobox patterning, hence, alterations in patterning may be leading to an effect on the cauda of the embryo. Among appendicular phenotypes specificity is ordered as stylopod, zeugopod, and then autopod. Interpreting the k-means plot for systemic developmental changes there is a visible progression to appendicular responses. The limb defects increase in frequency following cranial and postcranial axial phenotypic changes, indicating that the limb is more responsive when preceded by other phenotypic regions’ alterations. In examining the sensitivity of phenotypic regions, through the fipronil example it is evident that while limb defects have less specificity than cranial responses, appendicular phenotypes are more sensitive than cranial phenotypes when exposed to chemicals inducing any adverse appendicular outcomes. Notably, the retinoids of ATRA and retinol are activating through the biological system throughout the embryo with broad effects in a manner consistent with their status as positive controls.

Hierarchical clustering of skeletal defects highlighted the prevalence and type of phenotypic changes. In the hierarchical clustering of skeletal phenotypes (Figure 2B), the potency (lower concentrations of chemical compound results in AC_50s_ more potent than other chemicals at the same relative concentration) of retinol and ATRA are greatest in association with skeletal defects in every zone of interest. These findings reinforce the validity of the chemical compound selection process applied, since ATRA and retinol, two known positive controls on the ATRA pathway, were objectively identified *via* the workflow in Figure 1. The progression of skeletal defects in the hierarchical clustering is consistent with the parallel skeletal region k-means clustering. All clusters contain one or more axial phenotypes; this suggests that axial phenotypes are the most frequent fetal outcomes and possess the greatest specificity of the skeletal defects associated with the 48 chemicals. Clusters a, b, and c are predominantly thoracic cage defects, with eventual viscerocranial and unspecified defects occuring. The consistency of the unspecified defects fit the patterning associated with axial defects, suggesting they may be axial features. Cluster d starts with the consistent occurrence of defects of the zeugopod phenotype, all axial zones, and neurocranial and viscerocranial regions. This hierarchical cluster demonstrated the specific progression of skeletal defects as the sensitive thoracic cage, progression to appendicular (stylopod and zeugopod) and increasing occurrences of cauda and viscerocranium defects.

K-means clustering of targets on the ATRA signaling pathway identified target disruption related to the 48 chemicals of interest (Figure 4B). In PC1 moving right to left on the *x*-axis, the first quintile included those chemicals that activated RAR, RXR, and/or DR5 readouts only. The next quintile adds chemicals that also activated the retinol signaling pathway (RSP) assay (Tox21) node. Furthermore, PC1 and PC2 clearly delineate CYP surrogate biomarker, RARA, and DR5 as the most disrupted targets along the ATRA signaling pathway (Figure 4B). DR5 is the most sensitive target, with disruption occuring in all k-means quadrants to some degree (Figure 4B). PC2 provides potency ranking with chemicals having the least potency, but greatest specificity at the top of the *y*-axis; the greatest potency and broad range of abnormalities occurs moving down the *y*-axis (e.g., retinol, ATRA, etc.). Figure 3B’s hierarchical clustering demonstrates that CYP surrogate biomarker and DR5 are the most specific targets to be activated on the ATRA signaling pathway. The potency of chemicals associated with CYP surrogate biomarker appear to be most potent due to the greater degree of activation of CYP surrogate biomarker when compared to even DR5. The overall severity of CYP surrogate biomarker’ disruption, common occurrence of DR5 and RARA in the hierarchical clustering are consistent with the k-means clustering. Of particular interest in both the hierarchical and k-means clusterings there are chemical classification patterns. Organochlorine pesticides (e.g., endosulfan, endrin, lindane, pyridaben, etc) consistently activate DR5. Furthermore, azole fungicides (e.g., triazoles, imidazoles, pyrazoles) have broad association with activation of CYP surrogate biomarker, RARA, and DR5. Organotins biocides preferentially activated RXRs (e.g., triphenyltin, tributyltin hydroxide). Sulfurons (e.g., linuron, forchlorfenuron) consistently activate CYP surrogate biomarker. Chemicals with unspecified or broad categorizations (e.g., SAR 150640, asprin, iprione, etc.) regularly activated DR5, followed by CYP surrogate biomarker, then RARA, and activated the other molecular targets of interest with lower specificity.

### Literature & Datamining as basis for MIEs and putative AOPs (pAOPs)

**FIGURE 5:**
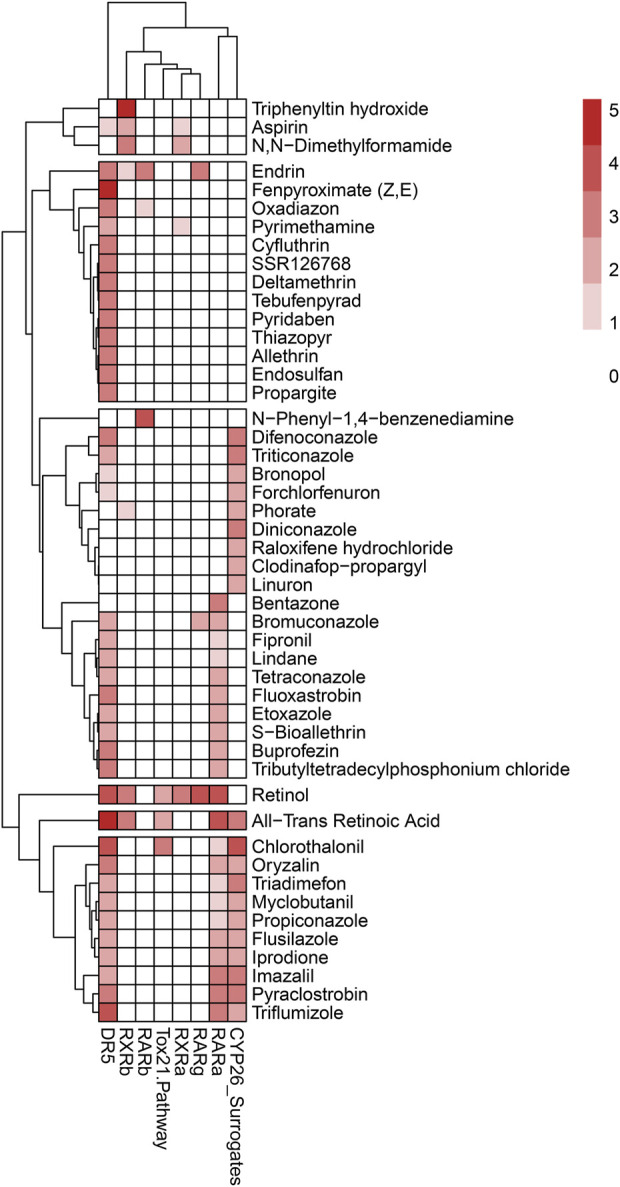
Heatmap dendrogram of 48 chemicals associated with ATRA pathway disruption and skeletal defects in ToxRefDB and ToxCast/Tox21 with potential AOP elucidation. Reflected are disruption activity of 8 targets of interest (RARA/B/G, RXRA/B, RSP (Tox21), DR5, and CYP surrogate biomarker). Scale of 0–5, with lightest color indicating less target disruption activity and darker red indicating higher target disruption activity compared to other chemicals for the respective targets. The dominance of activity at DR5, CYP surrogate biomarker, and RARA is consistent with k-means and hierarchical clustering diagrams; however, the heatmap attributes a greater amount of sensitivity of DR5 to chemicals of interest through the consistently darker red shading. CYP26 and RARA follow DR5 as the most disrupted of the ATRA signaling pathway targets.

To further support the formation of pAOPs, publicly available databases that describe *in vitro* assay results of a chemical’s activity against one of the ATRA pathway targets of interest were mined (Baker et al., 2022). Databases mined included Protein Data Bank (PDB) (Berman et al., 2003), ChEMBL (Gaulton et al., 2017), and ToxCast/Tox21 (Judson et al., 2016; Richard et al., 2016). Chemicals with inactivity in the ToxCast and Tox21 assays were excluded from the developed ATRA pathway candidate chemicals (Baker et al., 2022) selected from assays and literature. There were 1188 candidate chemicals (Baker et al., 2022) with activity in one or more ToxCast or Tox21 assays of interest. Furthermore, a multi-rule filter was set to limit chemicals with the following qualifying features: DR5 < 20 μM, CYP <10 μM, RAR <10 μM, RXR <10 μM, RSP (Tox21) < 10 μM, and prevalent RDH10 literature. This resulted in the shrinkage of data for retinoid disruption (potential MIEs) and adverse skeletal outcome (ASO) to 117 chemicals of particular interest for pAOP elucidation. Supplementary Figure S2 provides k-means clustering ATRA Signaling Pathway for 117 chemicals. Figure 5 is the heatmap dendrogram of 48 chemicals associated with ATRA pathway disruption and skeletal defects in ToxRefDB and ToxCast/Tox21, while S2 is a dendrogram of 117 chemicals with ATRA pathway disruption with potential AOP elucidation**.** For pAOPs, MIEs were derived from the measured target activity increases (agonist assays) and decreases (antagonist assays) in the Tox21 and ToxCast associated with the chemicals of interest. Adverse skeletal outcomes (ASOs) were based on the phenotypic defects recorded in ToxRefDB. The literature review conducted provided quantitative and qualitative data to inform key event (KE) placement in pAOPs. Table 3 displays 3 pAOPs for ASOs.

**TABLE 3:** | pAOPs for skeletal dysmorphogenesis linked to disruption of retinoid signaling. MIE, Molecular Initiating Event; KE, Key Events upstream to downstream; AO, Adverse Outcome. MIEs included loss of CYP26 enzymatic activity/expression and overactivation of RARs. KEs are critical imbalances to local ATRA concentration and FGF8 presence and signaling, modification of axial patterning genes, and cell death and differentiation. AOs are phenotypes resulting from stage and positional alterations in the fetal skeleton (Knudsen et al., 2021). MIEs were largely derived from quantitative data, while KEs were developed through the literature, and AOs were gleaned from a combination of literature and datamining.

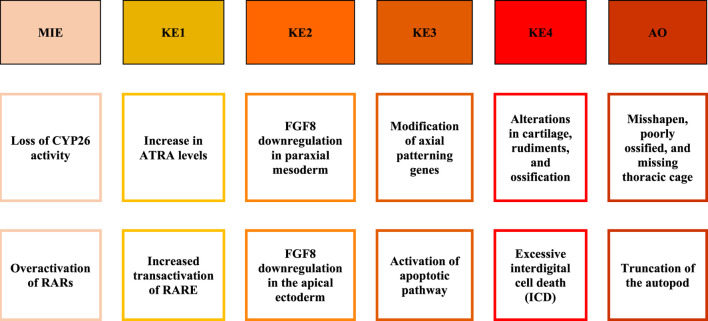

## Discussion

Forty-eight chemicals were found to represent a subset of the chemical landscape having *in vitro* (ATRA pathway targets) and *in vivo* (phenotypic skeletal defects) outcomes defined from ToxCast, Tox21, and ToxRefDB. K-means clustering, hierarchical clustering, and the heatmap results demonstrate the same conclusions about the 48 chemicals associated to both skeletal defects and target disruption on the ATRA signaling pathway. Thoracic cage was the most sensitive and frequently occurring skeletal defect in this model, followed by other axial defects (vertebra and cauda), and cranial defects. Simultaneously, DR5 has the greatest occurrence of target activity; chemicals disrupting DR5 were consistently associated with thoracic cage defects. Thereafter, CYP surrogate biomarker and RARA are the most sensitive targets to disruption on the ATRA signaling pathway. The most sensitive skeletal defects and disrupted targets outcomes are often associated with the same chemicals. The most potent chemicals are similar for both skeletal defects and target disruption (e.g., ATRA, retinol, flusilazole, N,N-dimethylformamide), increasing the likelihood that when disruption of a target on the ATRA signaling pathway results in an adverse outcome (AO) occurring with an associated ASO.

It should be noted that the workflow used in developing this model (Figure 1) was performed in series (supervised → unsupervised learning). Supervised learning culled specific defects (skeletal) and assay targets (ATRA system) from the datasets to localize the overlapping domain. From there, we used unsupervised methodology to classify statistical correlations. This led to inferences using only input vectors without referring to known, or labelled, outcomes for predictive toxicology (Ciallella et al., 2022); however, it neglects non-skeletal targets as well as chemicals that did not perturb *in vitro* bioactivity profiles of the ATRA assays available for this analysis, or non-ATRA pathways, which are important for health-protective inferences for DART testing (Rajagopal et al., 2022). The k-means clustering (Figure 4) findings for the 48 chemicals were highly relevant, and consistent with corresponding heatmap findings (Figure 5). However, for the 117 chemicals, the k-means clustering diagram is biased by the weight of the increased occurrence of Tox21 results, and lack of parallel ToxCast data (S2). This is evident with the straight-lined clustering of Tox21 data; while k-means clustering may not provide sufficient data about the relationships between target disruption on the ATRA signaling pathway, the 117 chemical heatmap (S2) findings are consistent with the 48 chemical heatmap (Figure 5). In both heatmaps Figure 5 and S2, DR5, CYP surrogate biomarker and RARA are highly perturbed by the chemicals of interest, however there is a notable increase in the RSP activity, likely due to the influx of Tox21 data, that was not complimented by equitable ToxCast data. The Tox21 RSP assay is especially related to RARA, and thus remains consistent with other data. These findings allude to the reality that an increase in data though increased Tox21 data may strengthen the conclusions of our findings. However, an increase in Tox21 assay types and number of chemicals in experimentation will lead to more data, require a complimentary increase in ToxCast data to achieve balanced and correctly scaled ToxPi results.

Twenty-eight of the 48 chemicals found both in ToxRefDB and Tox21 and/or ToxCast were candidate reference chemicals (Baker et al., 2022), but had not previously been noted. This finding provides reason for further exploration of the impact of the other 20 chemicals on the ATRA signaling pathway and skeletal defects. All 20 compounds were found to be associated with skeletal defects in literature (S1), but not ATRA signaling pathway disruption. For example, prenatal aspirin exposure in rats induces skeletal anomalies including fused ribs, incomplete ossification of the cervical arch, absent body of thoracic or lumbar vertebra, deformation of lumbar arch, and cartilaginous changes in vertebrae, paw and ribs (Dodo et al., 2009). There are 69 chemicals from the 117 chemicals that are lacking ToxRefDB data. This study focused on *in vivo* skeletal defects data from ToxRefDB; extending *in vivo* data to include data from other *in vivo* databases may show greater consistency between the 48 chemicals and 117 chemicals, the *in vivo* and *in vitro* data.

Chemical classification and structure may be associated with target disruption on the ATRA signaling pathway and skeletal defects based on our findings and those from the literature. Our analysis demonstrated that organochlorine pesticides (e.g., endosulfan, endrin, lindane, pyridaben, etc.) consistently activate DR5. Fungicides of the “-azoles” (e.g., triazoles, imidazoles, pyrazoles) family have broad association with activation of CYP surrogate biomarker, RARA, and DR5 (Figure 4). Organotin biocides preferentially activate RXRs (e.g., triphenyltin hydroxidetributyltin, tributyltetradecylphosphonium chloride). Phenylureas (e.g., linuron, forchlorfenuron) activate CYP surrogate biomarker. Compounds without common chemical classifications (e.g., SAR 150640, asprin, iprione, etc.) regularly activated DR5, followed by CYP surrogate biomarker, then RARA, and occasionally activated the other molecular targets of interest. The similar ToxPi outcomes between chemicals of the same class and comparable structures (analogue) provide evidence that these chemicals may be able to predict bioactivity information and skeletal defect outcomes for other similar compounds (Marvel et al., 2018). This “read-across” method would provide valuable predictive toxicological and hazard index information about chemicals without previous experimentation and at a faster rate than lab work.

We developed a computational model for predictive toxicology of 48 chemicals associated with chemical disruption of the ATRA signaling pathway with concurrent fetal skeletal defects. We found literature relationships between 48 chemicals and skeletal defects in various animal models (S1) consistent with our computational models. For example, mouse and several other animal models showed reductive limb defects resembling those seen in vitamin A deficiency (VAD); truncation and deletion of the long bones (ulna, radius, and humerus in the forelimb) and digital deletions and fusion (Kochhar, 1973, 1985). Retinoic acid administration to mothers produced severe, multiple skeletal defects and more specific malformations involving the axial skeleton, the fore- and hindlimbs, and cleft palate in embryos (Kistler, 1981). Embryonic exposure to pyrethroids causes craniofacial malformations, body axis curvature in zebrafish embryo model (DeMicco et al., 2010). Chlorothalonil (2,4,5,6-tetrachloro-1,3-benzenedicarbonitrile) is a broad-spectrum, non-systemic, organochlorine fungicide and exposure to this organochlorine fungicide causes skeletal malformations including absence of xiphoid process, malformation of the supraoccipital, absence of caudal vertebrae, incomplete ossification and malformation of xiphoid process in rats (Silva et al., 2020). Furthermore, five organochlorine pesticides, specifically, chlordane, endrin, and endosulfan, activate human RAR-mediated gene transcription *via* a retinoic acid response element (RARE such as DR5) and these organochlorine pesticides cause skeletal defects in several animal models (Ottolenghi et al., 1974; Lemaire et al., 2005; Kamata et al., 2008; Inoue et al., 2010). Moreover, organotin biocides preferentially activate RXRs and induced skeletal defects in animal models. For example, pregnant mice and rats exposed to triphenyltin hydroxidetributyltin and tributyltetradecylphosphonium chloride showed skeletal malformations including skeleton variations (e.g., poorly ossified skull bones) and malformations (misshapened axis and skull bones) an increased incidence of fusion of ribs, irregular ossification of sternebrae, and bipartite vertebral centers and cleft palate in mice and rat embryos (Sarpa et al., 2007).

Other classified chemicals from the 48 compounds such as phenylureas (e.g., linuron) activate CYP surrogate biomarker and induce skeletal abnormalities including abnormal axial rotation, and abnormal branchial arch development in rat embryo (McNutt and Harris, 1994), with linuron exposure to zebrafish models inducing spinal curvature and tail specific skeletal defects (Maharaj et al., 2020). Furthermore, CYP26A1(−/−) knockout mouse embryos having limited ATRA degradation had posterior elongation of axial patterning and displayed severe truncation of posterior structures (Rhinn and Dollé, 2012).

Several experimental studies have shown that retinoic acid inhibits chondrogenesis and that this inhibition is most likely RARA/B/G-mediated (Eckhardt and Schmitt, 1994; Schroeder et al., 1994). Additionally, studies have shown that an RARA-specific agonist is significantly more potent in inducing skeletal malformations more frequently than RARB- or RARG-specific agonists (Elmazar et al., 1996). RARG is abundantly expressed during chondrocyte maturation, whereas RARAs appears to be expressed in most other tissues of the embryonic limb development. Therefore, under conditions of excessive retinoid where expression of RARB (Mendelsohn et al., 1991; Jiang et al., 1994), RARA, and DR5 may be elevated within the limb mesenchyme region, and can lead to a delay or inhibition of chondrocytes and chondroblast differentiation, resulting in abnormal skeletogenesis. Furthermore, pregnant mice treated with commercially available RARA/B/G-ligands and ATRA in chronological sequences exhibited external, visceral and skeletal malformations by E18 of gestation. In these studies, teratogenic RAR-ligand potency rankings of RARA > RARB > RARG—these are consistent with RAR-ligands’ potency ranking found in our study. This study concluded that these retinoids also produced a different spectrum of developmental defects, specifically, the A-ligand and G-ligand induced the mandible, limb malformations, bone ossification deficiencies and defects of the sternebrae and vertebral body—with relative consistency among our compounds associated with these target disruptions and congruent skeletal defects (Elmazar et al., 1996).

The identification of RAREs (e.g., DR5) by RAR-binding assay in animal studies, *in vitro* cell line transfection assays, and ligand-binding transgenes reporter assay in mouse have been used to identify ATRA targeted genes that may be required for embryonic development (Cunningham and Duester, 2015). There were several studies that report the insufficiency or excess of ATRA due to specific RAREs (e.g., DR5) may lead to developmental defects in embryo (Houle et al., 2003; Kumar et al., 2016). DR5’s ability to influence a wide variety of skeletal defects is represented in our study as well as throughout the literature. For example, DR5, functioning as a RARE enhancer, can overactivate the Homeobox protein Hox-a1 (*Hoxa1*) gene, and disrupt hindbrain development (Langston and Gudas, 1992; Dupé et al., 1997; Houle et al., 2003; Pouilhe et al., 2007). Whereas, RARE that function as a silencer to repress Fibroblast growth factor-8 (*Fgf8*) expression in caudal region causes axial defects in embryo (Kumar et al., 2016). Hypoactivation of Transglutaminase 2 (*Tgm2*) by DR5 leads to abnormal interdigital limb development (Nagy et al., 1996; Dupé et al., 1999).

Chemical disruption in the ATRA signaling pathway can be linked to either elevated or diminished retinoid concentrations in target tissues, resulting either in up- or downregulation of RAR-mediated signaling, respectively. Under these circumstances, the model presented in the current manuscript covers a condition of only elevated ATRA concentration and upregulated RAR-signaling resulting from environmental exposure. In contrast, chemicals that disrupt retinol transport or ATRA bioactivation would be expected to invoke developmental phenotypes similar to Vitamin A deficiency (Knudsen et al., 2021). For example, a human biomonitoring study provided evidence associating reduction in circulating retinol levels with the adipose accumulation of persistent organic pollutants (POPs), including some polychlorinated biphenyl (PCB) congeners and dichlofol (Galbán-Velázquez et al., 2021). One mechanistic scenario suggested by those authors was chemical CYP oxidation and induction of an oxidative stress microenvironment that, in turn, lowers retinol levels by interfering with retinol binding protein-4 (RBP4) as a surrogate marker for serum retinol that forms a circulating carrier complex with transthyretin (TTR). The absence of HTS assays for retinol transport and metabolism is a limitation of the current model as it may apply to conditions associated with diminished retinoid concentrations and downregulated retinoid signaling.

Not all relevant biomarkers of retinol delivery were covered in this project. Retinoid delivery to the fetus is frequently mediated by RBP4, however, this is not the only pathway; postprandial retinoids delivered *via* chylomicrons is also an important pathway by which retinoids are delivered to fetuses (Steinhoff et al., 2021). This additional pathway illuminates the fact that there are multiple pathways of retinol delivery to the fetus, providing the occasion for others to study additional delivery methods through models such as found in this project.

Various CYPs with potential effects on ATRA metabolism could be altered in their expression by activation of AhR, CAR, and PXR pathways that, like RARs also heterodimerize with RXRs (McSorley and Daly, 2000; Esteban et al., 2021). The present study focused on biochemical (cell-free) CYP assays in ToxCast that, unfortunately, do not include CYP26 (this deficiency is currently being updated in the Tox21 portfolio).

Retinoids are inherent component of human and animal diets, with aquatic animals most exposed in early life stages (Kubickova et al., 2021). A broad range of retinoids are used to influence skin structure development cosmetically, as well as for use as dermatological, hematological, and infectious disease treatments (Zasada and Budzisz, 2019; Cosio et al., 2021). Environmental residues from chemicals such as the antifungal azoles may occur by applications on skin or environmentally through wastewater or discarded pharmaceuticals (Daughton and Ruhoy, 2009).

Socio‐economic factors have been found to influence maternal and fetal exposure to many chemical compounds (Renzo et al., 2015; Sprinkle and Payne-Sturges, 2021). A limitation of this study is high-throughput screening data compiled is at times derived from a limited set of not-diverse cell lines, resulting in a lack of understanding about the implications of our findings on populations that may be most affected by exposure to these chemical compounds. Greater diversity in original tissue and molecular samples would allow for greater direct applicability of study findings to more people, and further illuminate the extent of the impact of environmental factors on developmental toxicity in populations facing environmental injustice.

Heatmaps, k-means clusters, and hierarchical clusters provided clear quantitative associations between chemicals and skeletal defects and target disruption that inform AOP formation. Our pAOPs provide linear, organized, and biologically-relevant perspectives about MIEs determined by quantitative data that lead to literature derived KEs, and concludes with qualitative and quantitative literature and data derived ASOs (Table 3). However, AOPs are deterministic, resulting in less dynamic and vigorous conclusions. AOPs fail to reflect the biological complexity of the realm in which MIE, KE, and AO activities occur. As NAMs become more refined with increased data availability and defining fit-for-purpose criteria, their results will have greater dependability and applicability (Parish et al., 2020). Furthermore, ToxCast and Tox21 data’s gene score transformations aims to exclude non-specific activity due to cell stress and cytotoxicity, however, these factors may lead to skeletal developmental defects (Baker et al., 2020). At the same time, cell stress and cytotoxicity exclusion may have resulted in false positives due to global effects such as target bioactivity levels, which were still collected due to data collection methods. We propose the use of Agent Based Modeling (ABM), a dynamic *in silico* method that will test the effects of these chemicals on skeletal appendicular development. If the data, analytical tools, and AOPs developed here are consistent with ABM findings, they will provide substantial support for the conclusions reached. ABMs allow for high quality sensitivity analysis, further quantification of predictions, and generation of sound hypotheses formulation (Baker et al., 2020; Knudsen et al., 2020). Computational models also allow for a larger-scale evaluation of high-throughput screening/high content screening data and the ability to derive results that cannot be obtained experimentally (Knudsen et al., 2021).

## Data Availability

Publicly available datasets were analyzed in this study. This data can be found here: https://comptox.epa.gov/dashboard/ and https://github.com/USEPA/CompTox-ToxRefDB.

## Supplementary material

Supplementary Figure S2 ToxPi results for 117 chemicals with pAOPs associated with disruption of elements of RA signaling pathway. Genes of interest include RARA/B/C, RXR A/B, DR5, Tox21 Pathway, CYP surrogate biomarker. Most potent chemicals associated with the greatest amount of gene disruption have the largest ToxPis and highest ToxPi rank (48). K-means Clustering ATRA Signaling Pathway for 117 chemicals. Disrupted activity in the following targets is of interest: RARA/B/C, RXR A/B, DR5, RSP (Tox21), biomarker. The straight-lined clusters (blue and green clusters) are likely due to activity in RSP (Tox21), but lack of experimentation and complimentary assays with ToxCast. PC2 has greatest potency decreasing from top to bottom. In vitro ToxPis have RSP (Tox21) as most sensitive (larger slices) on the top, with decreasing sensitivity (smaller slices) on the bottom of the PC2. In the PCA plot there is an obvious effect on ToxPi composition, though there is more data needed for a fuller picture. The straight-lined clusters are likely due to activity in Tox21, but lack of experimentation with ToxCast. PCA2 is greatest potency from top to bottom. In vitro ToxPis have RSP (Tox21) as most sensitive (larger slices) on the top and to and decreasing sensitivity (smaller slices) on the bottom of the PCA2. In the PCA plot there is an obvious effect. However, the hierarchical clustering plot is not informative with these 117 chemicals. Heatmap dendrogram of 117 chemicals associated with ATRA pathway disruption and skeletal defects in ToxRefDB and ToxCast/Tox21 with potential AOP elucidation. Reflected are disruption activity of 8 targets of interest (RARA/B/G, RXRA/B, RSP (Tox21), DR5, and CYP surrogate biomarker) in association with 117 chemicals of interest. Scale of 0-5, with lightest color indicating less target disruption activity and darker red indicating higher target disruption activity compared to other chemicals for the respective targets. The heatmaps attributes a greater amount of sensitivity of DR5 to chemicals of interest through the consistently darker red shading. In order, DR5, CYP26, and then RARA are the most disrupted of the ATRA signaling pathway targets in the heatmap.

## References

[B1] Adams M. K. Belyaeva O. V. Wu L. Kedishvili N. Y. (2014). The retinaldehyde reductase activity of dhrs3 is reciprocally activated by retinol dehydrogenase 10 to control retinoid homeostasis. J. Biol. Chem. 289, 14868–14880. 10.1074/jbc.M114.552257 24733397 PMC4031538

[B2] Baker N. Boobis A. Burgoon L. Carney E. Currie R. Fritsche E. (2018). Building a developmental toxicity ontology. Birth Defects Res. 110, 502–518. 10.1002/bdr2.1189 29383852

[B3] Baker N. C. Pierro J. D. Taylor L. W. Knudsen T. B. (2022). Identifying candidate reference chemicals for *in vitro* testing of the retinoid pathway. Altex. 10.14573/altex.2202231 PMC10765368 35796328

[B4] Baker N. C. Sipes N. S. Franzosa J. Belair D. G. Abbott B. D. Judson R. S. (2020). Characterizing cleft palate toxicants using toxcast data, chemical structure, and the biomedical literature. Birth Defects Res. 112, 19–39. 10.1002/bdr2.1581 31471948 PMC8454266

[B5] Baker N. Knudsen T. Williams A. (2017). Abstract sifter: A comprehensive front-end system to PubMed. Chem Inf Sci. 6, 1–5. 10.12688/f1000research.12865.1 PMC5801564 29479422

[B6] Balmer J. E. Blomhoff R. (2005). A robust characterization of retinoic acid response elements based on a comparison of sites in three species. J. Steroid Biochem. Mol. Biol. 96, 347–354. 10.1016/j.jsbmb.2005.05.005 16081280

[B7] Berman H. Henrick K. Nakamura H. (2003). Announcing the worldwide protein data bank. Nat. Struct. Biol. 10, 980. 10.1038/nsb1203-980 14634627

[B8] Blaner W. S. Li Y. Brun P. J. Yuen J. J. Lee S. A. Clugston R. D. (2016). Vitamin a absorption, storage and mobilization. Subcell. Biochem. 81, 95–125. 10.1007/978-94-024-0945-1_4_4 27830502

[B9] Chambon P. (1994). The retinoid signaling pathway: Molecular and genetic analyses. Semin. Cell Biol. 5, 115–125. 10.1006/scel.1994.1015 8068884

[B10] Chawla B. Schley E. Williams A. L. Bohnsack B. L. (2016). Retinoic acid and pitx2 regulate early neural crest survival and migration in craniofacial and ocular development. Birth Defects Res. B Dev. Reprod. Toxicol. 107, 126–135. 10.1002/bdrb.21177 27175943

[B11] Chen Y. Sakamuru S. Huang R. Reese D. H. Xia M. (2016). Identification of compounds that modulate retinol signaling using a cell-based qhts assay. *Toxicol* . Toxicol. Vitro 32, 287–296. 10.1016/j.tiv.2016.01.011 PMC4779714 26820057

[B12] Ciallella H. L. Russo D. P. Sharma S. Li Y. Sloter E. Sweet L. (2022). Predicting prenatal developmental toxicity based on the combination of chemical structures and biological data. Environ. Sci. Technol. 56 (9), 5984–5998. 10.1021/acs.est.2c01040 35451820 PMC9191745

[B13] Collins F. S. Gray G. M. Bucher J. R. (2008). Toxicology. Transforming environmental health protection. Sci. (New York, N.Y.) 319, 906–907. 10.1126/science.1154619 PMC2679521 18276874

[B14] Cosio T. Gaziano R. Zuccari G. (2021). Retinoids in fungal infections: From bench to bedside. Pharmaceuticals 14 (10), 962. 10.3390/ph14100962 34681186 PMC8539705

[B15] Cunningham T. J. Duester G. (2015). Mechanisms of retinoic acid signalling and its roles in organ and limb development. Nat. Rev. Mol. Cell Biol. 16, 110–123. 10.1038/nrm3932 25560970 PMC4636111

[B16] Cunningham T. J. Zhao X. Sandell L. L. Evans S. M. Trainor P. A. Duester G. (2013). Antagonism between retinoic acid and fibroblast growth factor signaling during limb development. Cell Rep. 3, 1503–1511. 10.1016/j.celrep.2013.03.036 23623500 PMC3745640

[B17] Daughton C. G. Ruhoy I. S. (2009). Environmental footprint of pharmaceuticals: The significance of factors beyond direct excretion to sewers. Environ. Toxicol. Chem. 28 (12), 2495–2521. 10.1897/08-382.1 19382823

[B18] DeMicco A. Cooper K. R. Richardson J. R. White L. A. (2010). Developmental neurotoxicity of pyrethroid insecticides in zebrafish embryos. Toxicol. Sci. 113, 177–186. 10.1093/toxsci/kfp258 19861644 PMC2794336

[B19] Dodo T. Fukuta T. Uchida K. Mineshima H. Okuda Y. Okada F. (2009). A comparative investigation of fetal skeletal anomalies in rats induced by acetylsalicylic acid with single- and double-staining techniques. Regul. Toxicol. Pharmacol. 54, 308–313. 10.1016/j.yrtph.2009.05.014 19467286

[B20] Draut H. Liebenstein T. Begemann G. (2019). New insights into the control of cell fate choices and differentiation by retinoic acid in cranial, axial and caudal structures. Biomolecules 9, E860. 10.3390/biom9120860 31835881 PMC6995509

[B21] Dupé V. Davenne M. Brocard J. Dolle P. Mark M. Dierich A. (1997). *In vivo* functional analysis of the hoxa-1 3' retinoic acid response element (3'rare). Dev. Camb. Engl. 124, 399–410. 10.1242/dev.124.2.399 9053316

[B22] Dupé V. Ghyselinck N. B. Thomazy V. Nagy L. Davies P. J. Chambon P. (1999). Essential roles of retinoic acid signaling in interdigital apoptosis and control of bmp-7 expression in mouse autopods. Dev. Biol. 208, 30–43. 10.1006/dbio.1998.9176 10075839

[B23] Eckhardt K. Schmitt G. (1994). A retinoic acid receptor alpha antagonist counteracts retinoid teratogenicity *in vitro* and reduced incidence and/or severity of malformations *in vivo* . Toxicol. Lett. 70, 299–308. 10.1016/0378-4274(94)90124-4 8284797

[B24] Elmazar M. M. Reichert U. Shroot B. Nau H. (1996). Pattern of retinoid-induced teratogenic effects: Possible relationship with relative selectivity for nuclear retinoid receptors rar alpha, rar beta, and rar gamma. Teratology 53, 158–167. 10.1002/(SICI)1096-9926(199603)53:3<158::AID-TERA3>3.0.CO;2-0:3<158::AID-TERA3>3.0.CO;2-0 8761883

[B25] Esteban J. Sánchez-Pérez I. Hamscher G. Miettinen H. M. Korkalainen M. Viluksela M. (2021). Role of aryl hydrocarbon receptor (AHR) in overall retinoid metabolism: Response comparisons to 2, 3, 7, 8-tetrachlorodibenzo-p-dioxin (TCDD) exposure between wild-type and AHR knockout mice. Reprod. Toxicol. 101, 33–49. 10.1016/j.reprotox.2021.02.004 33607186

[B26] Foti R. S. Diaz P. Douguet D. (2016a). Comparison of the ligand binding site of cyp2c8 with cyp26a1 and cyp26b1: A structural basis for the identification of new inhibitors of the retinoic acid hydroxylases. J. Enzyme Inhib. Med. Chem. 31, 148–161. 10.1080/14756366.2016.1193734 PMC6628712 27424662

[B27] Foti R. S. Isoherranen N. Zelter A. Dickmann L. J. Buttrick B. R. Diaz P. (2016b). Identification of tazarotenic acid as the first xenobiotic substrate of human retinoic acid hydroxylase cyp26a1 and cyp26b1. J. Pharmacol. Exp. Ther. 357, 281–292. 10.1124/jpet.116.232637 26937021 PMC4851321

[B28] Galbán-Velázquez S. Esteban J. Çakmak G. Artacho-Cordon F. Leon J. Barril J. (2021). Associations of persistent organic pollutants in human adipose tissue with retinoid levels and their relevance to the redox microenvironment. Environ. Res. 195, 110764. 10.1016/j.envres.2021.110764 33497679 PMC8127078

[B29] Gaulton A. Hersey A. Nowotka M. Bento A. P. Chambers J. Mendez D. (2017). The chembl database in 2017. Nucleic Acids Res. 45, D945–D954. 10.1093/nar/gkw1074 27899562 PMC5210557

[B30] Ghyselinck N. B. Duester G. (2019). Retinoic acid signaling pathways. Dev. Camb. Engl. 146, 1–22. 10.1242/dev.167502 PMC6633611 31273085

[B31] Glineur R. Louryan S. Lemaître A. Evrard L. Rooze M. De Vos L. (1999). Cranio-facial dysmorphism: Experimental study in the mouse, clinical applications. Surg. Radiol. Anat. 21, 41–47. 10.1007/BF01635051 10370992

[B32] Grimm F. A. Iwata Y. Sirenko O. Chappell G. A. Wright F. A. Reif D. M. (2016). A chemical-biological similarity-based grouping of complex substances as a prototype approach for evaluating chemical alternatives. Green Chem. 18, 4407–4419. 10.1039/c6gc01147k 28035192 PMC5179981

[B33] Houle M. Sylvestre J. R. Lohnes D. (2003). Retinoic acid regulates a subset of cdx1 function *in vivo* . Dev. Camb. Engl. 130, 6555–6567. 10.1242/dev.00889 14660544

[B34] Huang R. Xia M. Sakamuru S. Zhao J. Shahane S. A. Attene-Ramos M. (2016). Modelling the tox21 10 k chemical profiles for *in vivo* toxicity prediction and mechanism characterization. Nat. Commun. 7, 10425. 10.1038/ncomms10425 26811972 PMC4777217

[B108] Hutson M. S. Leung M. C. K. Baker N. C. Spencer R. M. Knudsen T. B. (2017). Computational model of secondary palate fusion and disruption. Chem. Res. Toxicol. 30 (4), 9655–979. 10.1021/acs.chemrestox.6b00350 PMC6196717 28045533

[B35] Inoue D. Sei K. Ike M. (2010). Disruption of retinoic acid receptor signaling by environmental pollutants. J. Health Sci. 56, 221–230. 10.1248/jhs.56.221

[B36] Isoherranen N. Zhong G. (2019). Biochemical and physiological importance of the cyp26 retinoic acid hydroxylases. Pharmacol. Ther. 204, 107400. 10.1016/j.pharmthera.2019.107400 31419517 PMC6881548

[B37] Janesick A. Wu S. C. Blumberg B. (2015). Retinoic acid signaling and neuronal differentiation. Cell. Mol. Life Sci. 72, 1559–1576. 10.1007/s00018-014-1815-9 25558812 PMC11113123

[B38] Jiang H. Gyda M. Harnish D. C. Chandraratna R. A. Soprano K. J. Kochhar D. M. (1994). Teratogenesis by retinoic acid analogs positively correlates with elevation of retinoic acid receptor-beta 2 mrna levels in treated embryos. Teratology 50, 38–43. 10.1002/tera.1420500106 7974253

[B39] Judson R. Houck K. Martin M. Richard A. M. Knudsen T. B. Shah I. (2016). Analysis of the effects of cell stress and cytotoxicity on *in vitro* assay activity across a diverse chemical and assay space. Toxicol. Sci. 153, 409. 10.1093/toxsci/kfw148 27605417 PMC7297301

[B40] Kamata R. Shiraishi F. Nishikawa J. Yonemoto J. Shiraishi H. (2008). Screening and detection of the *in vitro* agonistic activity of xenobiotics on the retinoic acid receptor. Toxicol. Vitro. 22, 1050–1061. 10.1016/j.tiv.2008.01.002 18289828

[B41] Kastner P. Grondona J. M. Mark M. Gansmuller A. LeMeur M. Decimo D. (1994). Genetic analysis of rxr alpha developmental function: Convergence of rxr and rar signaling pathways in heart and eye morphogenesis. Cell 78, 987–1003. 10.1016/0092-8674(94)90274-7 7923367

[B42] Kistler A. (1981). Teratogenesis of retinoic acid in rats: Susceptible stages and suppression of retinoic acid-induced limb malformations by cycloheximide. Teratology 23, 25–31. 10.1002/tera.1420230106 7245088

[B44] Kleinstreuer N. C. Yang J. Berg E. L. Knudsen T. B. Richard A. M. Martin M. T. (2014). Phenotypic screening of the toxcast chemical library to classify toxic and therapeutic mechanisms. Nat. Biotechnol. 32, 583–591. 10.1038/nbt.2914 24837663

[B46] Knudsen T. B. Kleinstreuer N. C. (2011). Disruption of embryonic vascular development in predictive toxicology. Birth Defects Res. C Embryo Today. 93, 312–323. 10.1002/bdrc.20223 22271680

[B47] Knudsen T. B. Martin M. T. Kavlock R. J. Judson R. S. Dix D. J. Singh A. V. (2009). Profiling the activity of environmental chemicals in prenatal developmental toxicity studies using the U.S. EPA's ToxRefDB. Reprod. Toxicol. 28, 209–219. 10.1016/j.reprotox.2009.03.016 19446433

[B48] Knudsen T. B. Pierro J. D. Baker N. C. (2021). Retinoid signaling in skeletal development: Scoping the system for predictive toxicology. Reprod. Toxicol. 99, 109–130. 10.1016/j.reprotox.2020.10.014 33202217 PMC11451096

[B49] Knudsen T. B. Spencer R. M. Pierro J. D. Baker N. C. (2020). Computational biology and *in silico* toxicodynamics. Curr. Opin. Toxicol. 23-24, 119–126. 10.1016/j.cotox.2020.11.001 PMC9770085 36561131

[B50] Kochhar D. M. (1973). Limb development in mouse embryos. I. Analysis of teratogenic effects of retinoic acid. Teratology 7, 289–298. 10.1002/tera.1420070310 4807131

[B51] Kochhar D. M. (1985). Skeletal morphogenesis: Comparative effects of a mutant gene and a teratogen. Prog. Clin. Biol. Res. 171, 267–281. 3157194

[B52] Kubickova B. Ramwell C. Hilscherova K. Jacobs M. N. (2021). Highlighting the gaps in hazard and risk assessment of unregulated endocrine active substances in surface waters: Retinoids as a European case study. Environ. Sci. Eur. 33, 20. 10.1186/s12302-020-00428-0

[B53] Kumar S. Cunningham T. J. Duester G. (2016). Nuclear receptor corepressors ncor1 and ncor2 (smrt) are required for retinoic acid-dependent repression of fgf8 during somitogenesis. Dev. Biol. 418, 204–215. 10.1016/j.ydbio.2016.08.005 27506116 PMC5031541

[B54] Lampen A. Meyer S. Arnhold T. Nau H. (2000). Metabolism of vitamin a and its active metabolite all-trans-retinoic acid in small intestinal enterocytes. J. Pharmacol. Exp. Ther. 295, 979–985. 11082432

[B55] Langston A. W. Gudas L. J. (1992). Identification of a retinoic acid responsive enhancer 3' of the murine homeobox gene hox-1.6. Mech. Dev. 38, 217–227. 10.1016/0925-4773(92)90055-o 1360810

[B56] Lautenberg Chemical Safety for the 21st Century Act (2016). 15 USC 2601, public Law 114-182. Available at: https://www.congress.gov/114/plaws/publ182/PLAW-114publ182.pdf .

[B57] Lee L. M. Leung C. Y. Tang W. W. Choi H. L. Leung Y. C. McCaffery P. J. (2012). A paradoxical teratogenic mechanism for retinoic acid. Proc. Natl. Acad. Sci. U. S. A. 109, 13668–13673. 10.1073/pnas.1200872109 22869719 PMC3427051

[B58] Lemaire G. Balaguer P. Michel S. Rahmani R. (2005). Activation of retinoic acid receptor-dependent transcription by organochlorine pesticides. Toxicol. Appl. Pharmacol. 202, 38–49. 10.1016/j.taap.2004.06.004 15589975

[B59] Leung M. C. Hutson M. S. Seifert A. W. Spencer R. M. Knudsen T. B. (2016). Computational modeling and simulation of genital tubercle development. Reprod. Toxicol. 64, 151–161. 10.1016/j.reprotox.2016.05.005 27180093

[B60] Maharaj S. El Ahmadie N. Rheingold S. El Chehouri J. Yang L. Souders C. L. 2nd (2020). Sub-lethal toxicity assessment of the phenylurea herbicide linuron in developing zebrafish (danio rerio) embryo/larvae. Neurotoxicol. Teratol. 81, 106917. 10.1016/j.ntt.2020.106917 32712134

[B61] Mark M. Ghyselinck N. B. Chambon P. (2009). Function of retinoic acid receptors during embryonic development. Nucl. Recept. Signal. 7, e002. 10.1621/nrs.07002 19381305 PMC2670431

[B62] Mark M. Ghyselinck N. B. Chambon P. (2006). Function of retinoid nuclear receptors: Lessons from genetic and pharmacological dissections of the retinoic acid signaling pathway during mouse embryogenesis. Annu. Rev. Pharmacol. Toxicol. 46, 451–480. 10.1146/annurev.pharmtox.46.120604.141156 16402912

[B63] Marty M. S. Andrus A. K. Groff K. A. (2022). Animal metrics: Tracking contributions of new approach methods to reduced animal use. ALTEX 39, 95–112. 10.14573/altex.2107211 34676883

[B64] Marvel S. W. To K. Grimm F. A. Wright F. A. Rusyn I. Reif D. M. (2018). Toxpi graphical user interface 2.0: Dynamic exploration, visualization, and sharing of integrated data models. BMC Bioinforma. 19, 80. 10.1186/s12859-018-2089-2 PMC5838926 29506467

[B65] McNutt T. L. Harris C. (1994). Lindane embryotoxicity and differential alteration of cysteine and glutathione levels in rat embryos and visceral yolk sacs. Reprod. Toxicol. 8, 351–362. 10.1016/0890-6238(94)90051-5 7524828

[B66] McSorley L. C. Daly A. K. (2000). Identification of human cytochrome P450 isoforms that contribute to all-trans-retinoic acid 4-hydroxylation. Biochem. Pharmacol. 60 (4), 517–526. 10.1016/s0006-2952(00)00356-7 10874126

[B67] Mendelsohn C. Ruberte E. LeMeur M. Morriss-Kay G. Chambon P. (1991). Developmental analysis of the retinoic acid-inducible rar-beta 2 promoter in transgenic animals. Dev. Camb. Engl. 113, 723–734. 10.1242/dev.113.3.723 1668276

[B68] Menegola E. Veltman C. H. J. Battistoni M. Di Renzo F. Moretto A. Metruccio F. (2021). An adverse outcome pathway on the disruption of retinoic acid metabolism leading to developmental craniofacial defects. Toxicology 458, 152843. 10.1016/j.tox.2021.152843 34186166

[B69] Metzler M. A. Sandell L. L. (2016). Enzymatic metabolism of vitamin a in developing vertebrate embryos. Nutrients 8, E812. 10.3390/nu8120812 27983671 PMC5188467

[B70] Mezquita B. Mezquita C. (2019). Two opposing faces of retinoic acid: Induction of stemness or induction of differentiation depending on cell-type. Biomolecules 9, E567. 10.3390/biom9100567 31590252 PMC6843238

[B71] Nagy L. Saydak M. Shipley N. Lu S. Basilion J. P. Yan Z. H. (1996). Identification and characterization of a versatile retinoid response element (retinoic acid receptor response element-retinoid x receptor response element) in the mouse tissue transglutaminase gene promoter. J. Biol. Chem. 271, 4355–4365. 10.1074/jbc.271.8.4355 8626785

[B72] National Academy of Sciences. A framework to guide selection of chemical Alternatives.National Academy of sciences. A framework to guide selection of chemical alternatives (2014). ISBN 978-0-309-31013-0

[B73] USEPA (2021). New Approach Methods Work Plan (v2). Washington, DC: U.S. Environmental Protection Agency. EPA/600/X-21/209.

[B74] Niederreither K. Dollé P. (2008). Retinoic acid in development: Towards an integrated view. Nat. Rev. Genet. 9, 541–553. 10.1038/nrg2340 18542081

[B75] OECD (2021). Detailed review paper on the retinoid system. Available at: https://www.oecd.org/officialdocuments/publicdisplaydocumentpdf/?cote=ENV-CBC-MONO(2021)20%20&doclanguage=en .

[B76] OECD (2014). Guidance document on standardised test guidelines for evaluating chemicals for endocrine disruption. Available at: https://www.oecd-ilibrary.org/content/publication/9789264221413-en.

[B77] Organisation for Economic Co-operation and Development (OECD) (2012). Detailed review paper on the state of the science on novel *in vitro* and *in vivo* screening and testing methods and endpoints for evaluating endocrine disruptors.

[B78] Ottolenghi A. D. Haseman J. K. Suggs F. (1974). Teratogenic effects of aldrin, dieldrin, and endrin in hamsters and mice. Teratology 9, 11–16. 10.1002/tera.1420090104 4204659

[B79] Parish S. T. Aschner M. Casey W. Corvaro M. Embry M. R. Fitzpatrick S. (2020). An evaluation framework for new approach methodologies (NAMs) for human health safety assessment. Regul. Toxicol. Pharmacol. 112, 104592. 10.1016/j.yrtph.2020.104592 32017962

[B80] Pouilhe M. Gilardi-Hebenstreit P. Desmarquet-Trin Dinh C. Charnay P. (2007). Direct regulation of vhnf1 by retinoic acid signaling and maf-related factors in the neural tube. Dev. Biol. 309, 344–357. 10.1016/j.ydbio.2007.07.003 17669392

[B81] Qin F. Shen Z. Peng L. Wu R. Hu X. Zhang G. (2014). Metabolic characterization of all-trans-retinoic acid (atra)-induced craniofacial development of murine embryos using *in vivo* proton magnetic resonance spectroscopy. Plos one 9, e96010. 10.1371/journal.pone.0096010 24816763 PMC4015972

[B82] Rajagopal R. Baltazar M. T. Carmichael P. L. Dent M. P. Head J. Li H. (2022). Beyond AOPs: A mechanistic evaluation of NAMs in DART testing. Front. Toxicol. 4, 838466. 10.3389/ftox.2022.838466 35295212 PMC8915803

[B83] Reif D. M. Martin M. T. Tan S. W. (2010). Endocrine profiling and prioritization of environmental chemicals using ToxCast data. Environ. Health Perspect. 118. 14–20. 10.1289/ehp.1002180 PMC3002190 20826373

[B84] Renzo Di Conry J. A. Blake J. DeNicola N. Martin J. N. Jr (2015). International federation of gynecology and obstetrics opinion on reproductive health impacts of exposure to toxic environmental chemicals. Int. J. Gynaecol. Obstet. 131, 219–225. 10.1016/j.ijgo.2015.09.002 26433469 PMC6663094

[B85] Rhinn M. Dollé P. (2012). Retinoic acid signalling during development. Dev. Camb. Engl. 139, 843–858. 10.1242/dev.065938 22318625

[B86] Richard A. M. Judson R. S. Houck K. A. Grulke C. M. Volarath P. Thillainadarajah I. (2016). Toxcast chemical landscape: Paving the road to 21st century toxicology. Chem. Res. Toxicol. 29, 1225–1251. 10.1021/acs.chemrestox.6b00135 27367298

[B87] Roberts C. (2020). Regulating retinoic acid availability during development and regeneration: The role of the cyp26 enzymes. J. Dev. Biol. 8, E6. 10.3390/jdb8010006 32151018 PMC7151129

[B88] Sarpa M. De-Carvalho R. R. Delgado I. F. Paumgartten F. J. R. (2007). Developmental toxicity of triphenyltin hydroxide in mice. Regul. Toxicol. Pharmacol. 49, 43–52. 10.1016/j.yrtph.2007.05.006 17619067

[B89] Schroeder V. Hashimoto Y. Heersche J. N. (1994). The effects of natural and synthetic retinoids on the differentiation of rcj c5.18 chondrogenic cells. Teratology 50, 54–62. 10.1002/tera.1420500108 7974255

[B90] Schubert M. Gibert Y. (2020). Retinoids in embryonic development. Biomolecules 10, E1278. 10.3390/biom10091278 32899684 PMC7564826

[B91] See A. W. Kaiser M. E. White J. C. Clagett-Dame M. (2008). A nutritional model of late embryonic vitamin a deficiency produces defects in organogenesis at a high penetrance and reveals new roles for the vitamin in skeletal development. Dev. Biol. 316, 171–190. 10.1016/j.ydbio.2007.10.018 18321479

[B92] Shannon S. R. Moise A. R. Trainor P. A. (2017). New insights and changing paradigms in the regulation of vitamin a metabolism in development. WIREs Dev. Biol. 6, 1–28. 10.1002/wdev.264 PMC5911347 28207193

[B93] Shannon S. R. Yu J. Defnet A. E. (2020). Chapter fifteen - identifying vitamin a signaling by visualizing gene and protein activity, and by quantification of vitamin a metabolites. in Methods in enzymology. Editor Pohl E. (Academic Press). 10.1016/bs.mie.2020.03.011 PMC7565286 32359653

[B94] Shenefelt R. E. (1972). Morphogenesis of malformations in hamsters caused by retinoic acid: Relation to dose and stage at treatment. Teratology 5, 103–118. 10.1002/tera.1420050115 5014447

[B95] Shimozono S. Iimura T. Kitaguchi T. Higashijima S. I. Miyawaki A. (2013). Visualization of an endogenous retinoic acid gradient across embryonic development. Nature 496, 363–366. 10.1038/nature12037 23563268

[B96] Silva J. N. D. Monteiro N. R. Antunes P. A. Favareto A. P. A. (2020). Maternal and developmental toxicity after exposure to formulation of chlorothalonil and thiophanate-methyl during organogenesis in rats. An. Acad. Bras. Cienc. 92, e20191026. 10.1590/0001-3765202020191026 33206784

[B97] Sipes N. S. Martin M. T. Reif D. M. Kleinstreuer N. C. Judson R. S. Singh A. V. (2011). Predictive models of prenatal developmental toxicity from ToxCast high-throughput screening data. Toxicol. Sci. 124, 109–127. 10.1093/toxsci/kfr220 21873373

[B98] Sprinkle R. H. Payne-Sturges D. C. (2021). Mixture toxicity, cumulative risk, and environmental justice in United States federal policy, 1980–2016. Environ. Health 20, 104–125. 10.1186/s12940-021-00764-5 34535123 PMC8449500

[B99] Steinhoff J. S. Lass A. Schupp M. (2021). Biological functions of RBP4 and its relevance for human diseases. Front. Physiol. 12 (659977), 1–15. 10.3389/fphys.2021.659977 PMC8006376 33790810

[B100] Teletin M. Vernet N. Ghyselinck N. B. Mark M. (2017). Roles of retinoic acid in germ cell differentiation. Curr. Top. Dev. Biol. 125, 191–225. 10.1016/bs.ctdb.2016.11.013 28527572

[B101] Thomas R. S. Bahadori T. Buckley T. J. Cowden J. Deisenroth C. Dionisio K. L. (2019). The next generation blueprint of computational toxicology at the u.S. Environmental protection agency. *Toxicological sciences* :. Toxicol. Sci. 169, 317–332. 10.1093/toxsci/kfz058 30835285 PMC6542711

[B102] Watford S. Ly Pham L. Wignall J. Shin R. Martin M. T. Friedman K. P. (2019). Toxrefdb version 2.0: Improved utility for predictive and retrospective toxicology analyses. Reprod. Toxicol. 89, 145–158. 10.1016/j.reprotox.2019.07.012 31340180 PMC6944327

[B103] Williams A. L. Bohnsack B. L. (2019). What's retinoic acid got to do with it? Retinoic acid regulation of the neural crest in craniofacial and ocular development. Genesis 57, e23308. 10.1002/dvg.23308 31157952

[B109] USEPA (2021). New Approach Methods Work Plan (v2). U.S. Environmental Protection Agency, Washington, DC. EPA/600/X-21/209

[B104] Yousefi B. Azizzadeh F. (2010). The histopathalogical effects of retinoic acid on the tissues. Pak. J. Biol. Sci. 13 (19), 927–936. 10.3923/pjbs.2010.927.936 21313915

[B105] Zasada M. Budzisz E. (2019). Retinoids: Active molecules influencing skin structure formation in cosmetic and dermatological treatments. Postepy Dermatol. Alergol. 36 (4), 392–397. 10.5114/ada.2019.87443 31616211 PMC6791161

[B106] Zhang T. G. Li X. D. Yu G. Y. Xie P. Wang Y. G. Liu Z. Y. (2015). All-trans-retinoic acid inhibits chondrogenesis of rat embryo hindlimb bud mesenchymal cells by downregulating p53 expression. Mol. Med. Rep. 12, 210–218. 10.3892/mmr.2015.3423 25738595 PMC4438916

[B107] Zurlinden T. J. Saili K. S. Rush N. Kothiya P. Judson R. S. Houck K. A. (2020). Profiling the ToxCast library with a pluripotent human (H9) stem cell line-based biomarker assay for developmental toxicity. Toxicol. Sci. 174, 189–209. 10.1093/toxsci/kfaa014 32073639 PMC8527599

