# Supplementary figures and images for: Computational model for fetal skeletal defects potentially linked to disruption of retinoic acid signaling
Source: Front Pharmacol. 2022 Sep 6;13:971296. doi: 10.3389/fphar.2022.971296 (PMC9511990; doi:10.3389/fphar.2022.971296)

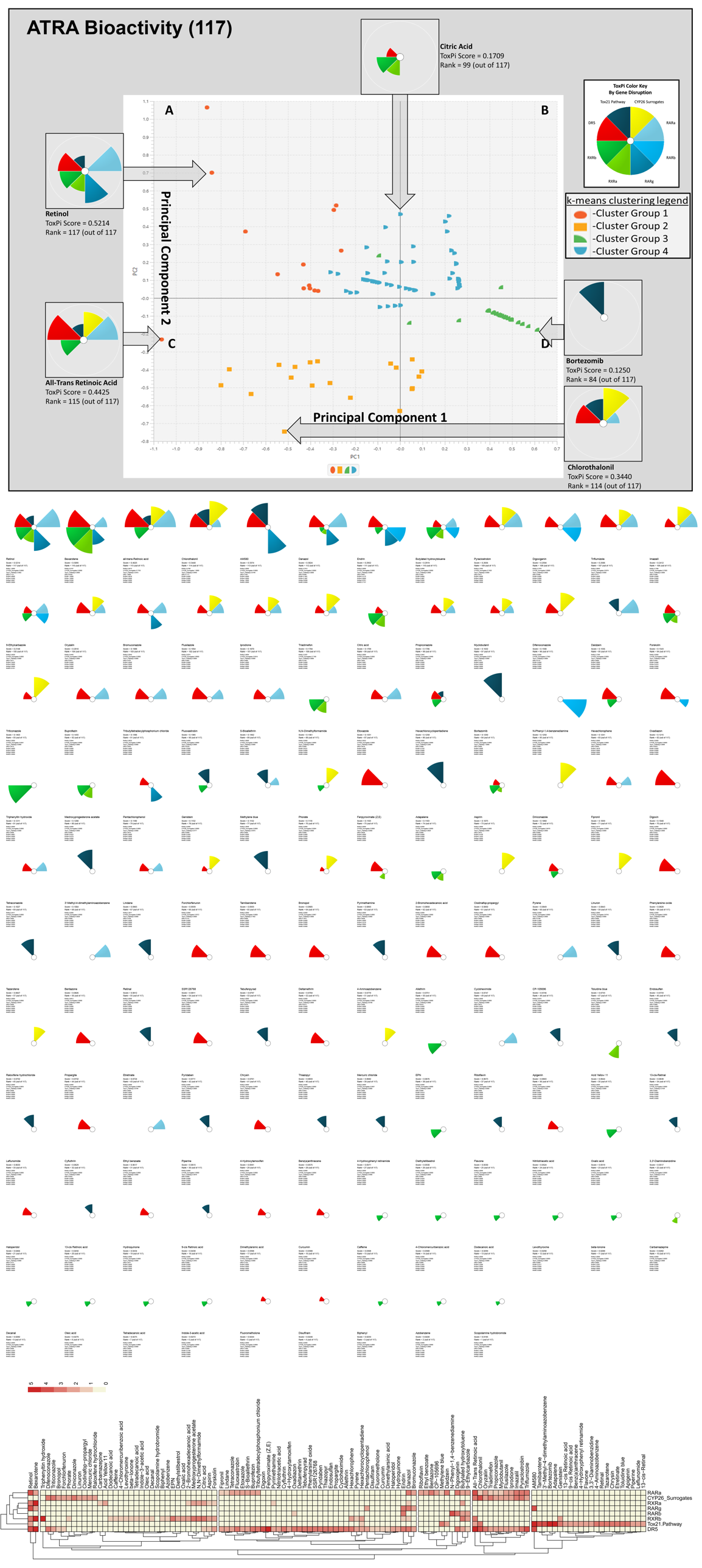

Supplement: Supplementary file 1 [file Image1.tif]
